# Evaluating implementation of a fire-prevention injury prevention briefing in children's centres: Cluster randomised controlled trial

**DOI:** 10.1371/journal.pone.0172584

**Published:** 2017-03-24

**Authors:** Toity Deave, Adrian Hawkins, Arun Kumar, Mike Hayes, Nicola Cooper, Michael Watson, Joanne Ablewhite, Carol Coupland, Alex Sutton, Gosia Majsak-Newman, Lisa McDaid, Trudy Goodenough, Kate Beckett, Elaine McColl, Richard Reading, Denise Kendrick

**Affiliations:** 1 Centre for Child & Adolescent Health, Health & Applied Sciences, University of the West of England Bristol, Bristol, United Kingdom; 2 Institute of Health & Society, Baddiley-Clark Building, Newcastle University, Newcastle upon Tyne, United Kingdom; 3 Division of Primary Care, School of Medicine, University of Nottingham, Nottingham, United Kingdom; 4 Child Accident Prevention Trust, Barnet, London, United Kingdom; 5 Department of Health Sciences, University of Leicester, Leicester, United Kingdom; 6 Faculty of Medicine and Health Sciences, School of Health Sciences, University of Nottingham, Nottingham, United Kingdom; 7 Norfolk and Suffolk Primary and Community Care Research Office, Hosted by South Norfolk CCG, Norwich, United Kingdom; 8 Norfolk and Norwich University Hospital, NHS Clinical Research and Trials Unit, Norwich Medical School, University of East Anglia, Norwich, United Kingdom; 9 University of the West of England, Research and Innovation, University Hospitals Bristol NHS Foundation Trust, Education Centre, Bristol, United Kingdom; 10 Newcastle Clinical Trials Unit, Newcastle University, Newcastle upon Tyne, United Kingdom; 11 Jenny Lind Paediatric Department, Norfolk and Norwich University Hospital, Norwich, United Kingdom; Monash University, AUSTRALIA

## Abstract

**Background:**

Many developed countries have high mortality rates for fire-related deaths in children aged 0–14 years with steep social gradients. Evidence-based interventions to promote fire safety practices exist, but the impact of implementing a range of these interventions in children’s services has not been assessed. We developed an Injury Prevention Briefing (IPB), which brought together evidence about effective fire safety interventions and good practice in delivering interventions; plus training and facilitation to support its use and evaluated its implementation.

**Methods:**

We conducted a cluster randomised controlled trial, with integrated qualitative and cost-effectiveness nested studies, across four study sites in England involving children’s centres in disadvantaged areas; participants were staff and families attending those centres. Centres were stratified by study site and randomised within strata to one of three arms: IPB plus facilitation (IPB+), IPB only, usual care. IPB+ centres received initial training and facilitation at months 1, 3, and 8. Baseline data from children’s centres were collected between August 2011 and January 2012 and follow-up data were collected between June 2012 and June 2013. Parent baseline data were collected between January 2012 and May 2012 and follow-up data between May 2013 and September 2013. Data comprised baseline and 12 month parent- and staff-completed questionnaires, facilitation contact data, activity logs and staff interviews. The primary outcome was whether families had a plan for escaping from a house fire. Treatment arms were compared using multilevel models to account for clustering by children’s centre.

**Results:**

1112 parents at 36 children’s centres participated. There was no significant effect of the intervention on families’ possession of plans for escaping from a house fire (adjusted odds ratio (AOR) IPB only vs. usual care: 0.93, 95%CI 0.58, 1.49; AOR IPB+ vs. usual care 1.41, 95%CI 0.91, 2.20). However, significantly more families in the intervention arms reported more behaviours for escaping from house fires (AOR IPB only vs. usual care: 2.56, 95%CI 01.38, 4.76; AOR IPB+ vs. usual care 1.78, 95%CI 1.01, 3.15).

**Conclusion:**

Our study demonstrated that children’s centres can deliver an injury prevention intervention to families in disadvantaged communities and achieve changes in home safety behaviours.

## Introduction

Childhood fire-related deaths and injuries are an important global issue[1] and are one of the leading causes of deaths for children under 14 years in the US.[2] In 2009, almost 119,000 U.S. children were injured severely enough due to unintentional fires and burns that they had to visit an ED. Fires and burns are one of the major causes of nonfatal unintentional injuries in children in the US. [2] Compared with other high-income countries, the UK has high mortality rates for deaths from fire and flames in children aged 0–14 years with steep social gradients in mortality.[1, 3, 4]

Some interventions are effective in reducing the risk of fire-related injury and in promoting fire-prevention practices.[5–8] Smoke alarms can reduce the risk of death in house fires.[5, 6] Providing education and smoke alarms can increase the prevalence of working smoke alarms and educational interventions can increase the prevalence of plans for escaping from house fires.[7, 8] Although evidence-based interventions to promote fire safety practices exist, translating research findings into practice does not always occur. Injury prevention programmes are unlikely to achieve their aims if they are not effectively implemented. Knowing how effective interventions can be implemented more widely is a major challenge in injury prevention. To address this translational gap, we developed a fire prevention intervention comprising an Injury Prevention Briefing (IPB)[9] that combined guidance on best practice for delivering injury prevention programmes in a real-world setting with evidence of effectiveness of interventions, along with training and a facilitation package to support implementation of the IPB.

The IPB was designed for use by children’s services such as children’s centres in the UK or Head Start programmes in the US. These services aim to improve outcomes for young children and to reduce inequalities in health, with a particular focus on the most disadvantaged.[10–12] They provide community-based services, information and support for families. Children’s centres focus on those with pre-school children, similar to those in Head Start, a US government-funded programme that serves low income families with children between 3-5years.[13] The services include home safety interventions and serve populations at particular risk of fire-related injury. However, the effectiveness of delivering a range of fire prevention activities, such as those included in the IPB in such settings is unknown. In this paper, we report on the evaluation of the effectiveness and cost effectiveness of implementing the IPB in children’s centres in the UK.

## Methods

### Design and setting

We undertook a three-arm multi-centre cluster randomised controlled trial (RCT), with integrated economic evaluation, in four sites in England (Nottingham, Bristol, Norwich and Newcastle). We used a cluster RCT to prevent contamination between families who attended the same children’s centre and because intervention delivery was more pragmatic at children’s centre level. Embedded qualitative interviews with key children’ staff at these sites provided additional contextual information regarding facilitators and barriers to IPB implementation.[14] Full details of the methods used[15] and of the qualitative analysis[14] are reported elsewhere.

### Participants

Children’s centres were invited to participate if their catchment area had more than 50% of under-5 year-olds living in one of the 30% most disadvantaged super output areas in England, which are geographical areas for the collection and publication of small area statistics.[16] Recruitment took place between 03/08/2011 and 10/01/2012.

Families living in the children’s centre catchment area, who had attended the centre in the previous three months with parents aged at least 16 years and a child under three years, were eligible. Recruitment took place between 05/01/2012 and 31/05/2012. The recruitment strategies are described elsewhere.[15, 17] For both children’s centres and families, recruitment was defined as providing written consent and completion of baseline questionnaire. All participants completed a consent form.

### Intervention

The intervention was developed using the UK Medical Research Council (MRC) guidance for the development and evaluation of complex interventions[18] and included identifying the evidence-base, identifying appropriate theory and modelling processes and outcomes. Evidence about the effectiveness of interventions was ascertained from a systematic review of interventions to prevent home injuries, including those from house fires[7] and a systematic review of facilitators and barriers for home injury prevention interventions for pre-school children.[19] Evidence about the design, content and delivery of the intervention came from several sources, including the Health Development Agency ‘Effective Action Briefing’ for putting evidence into practice for the promotion of domestic smoke alarms[20] and a review of reviews of literature on the implementation and facilitation of health promotion interventions, undertaken as preliminary work for this trial. We also interviewed national and local leaders, undertook workshops, one in each trial site, with community practitioners, staff in children’s centres, from the Fire and Rescue Service, NHS and commissioners. By doing this, we brought together the scientific evidence on what works, with best practice from those who deliver injury prevention programmes in the community.

A number of common themes emerged from the review of reviews; four reviews had convergent findings about the explanatory factors that affect the implementation of community prevention programmes; one of which was training and technical assistance.[21–24] However national guidance documents, such as those from the National Institute for Care Excellence, are often provided in the UK without such support. The trial therefore comprised two intervention arms, one where the IPB was provided with training and facilitation (described below) the other provided the IPB without training and facilitation.

The intervention was developed based on five behavioural change theories (health belief model, social cognitive theory, theory of reasoned action, theory of self-regulation and self-control and theory of subjective culture and interpersonal relations) from a review of behaviour change theories for injury prevention.[25] Our intervention aimed to address the three factors described as necessary and sufficient for producing a behaviour change by helping participants (both children’s centre staff and families) form intentions to change behaviour, remove environmental barriers and provide participants with the knowledge and skills to perform the behaviour.

There were two intervention arms and one usual care arm:

1. IPB delivery, a three hour staff training session and on-going facilitation to support implementation (IPB+);
2. IPB posted to children’s centre (IPB only);
3. Children’s centre undertake usual fire prevention activity (usual care).

The IPB was developed specifically for use in children’s centres[9] using a seven-step process.[20, 26] The IPB provided advice, information and activities to support delivery of five key fire safety messages: smoke alarm use and maintenance, plans for escaping from a house fire, potential causes of house fires, safe storage of matches and lighters and bedtime fire safety routines. The training provided information on fire-related injuries; development, principles and content of the IPB; practice in using the IPB and in the development of the IPB implementation plan.

The facilitation comprised telephone or face-to-face contacts from the research team at 1, 3 and 8 months to collect information on implementation progress, address questions and barriers to implementation and provide advice, examples of good practice, a resource list and contacts with other organisations, e.g., fire and rescue service. Children’s centres vary considerably in their management and operational processes and in the populations they serve but they are experienced in delivering health promotion programmes. For this reason, and to ensure the implementation of the IPB reflected the real-world setting, they were asked to develop a plan for implementing the IPB which was most suitable to their circumstances and those of the families they serve. If they were unable to deliver all five fire-safety messages, they were asked to focus on smoke alarms and fire escape plans as these have the strongest evidence-base. Children’s centres used their usual centre processes for disseminating information about the IPB to staff, both current and new.

### Outcomes

The primary outcome was a family level binary variable for whether or not the family had a plan for escaping from a house fire. Secondary outcomes are described in Box 1:

**Box 1. Secondary outcomes**

**Family outcomes**

1 Intervention family reported more fire escape behaviours than those families in the control group (using a binary measure derived from five component items using latent variable analysis. The component items are: having door keys accessible, having window lock keys accessible, having a torch beside the bed, knowing the sound of a smoke alarm and having exits clear);
2 Family had smoke alarms fitted and working on every level of their home;
3 Family reported fire-setting or match play by their children;
4 Family reported bedtime fire safety routine score;
5 Family had accessed smoking cessation services;
6 The number of correct responses to fire safety knowledge questions;
7 Family reported being fairly satisfied or very satisfied with home safety information provided by children’s centres;
8 Implementation of the IPB assessed by:
  a Family had received advice on each of the 5 key messages in the IPB in the last year;
  b Family had attended a fire safety session in the last year;
  c The number of fire safety sessions attended by family in the last year;
  d Family had attended a fire safety session at a children’s centre in the last year;
  e Family had attended sessions about each of the 5 key messages in the IPB in the last year;
9 Family’s resource-use and expenditure in relation to fire safety practices.

**Children’s centre outcomes**

10 Children’s centre provided information and advice on fire prevention;
11 Resource use and expenditure incurred in relation to fire prevention activities
12 Reported implementation of the IPB within children’s centres;
13 Barriers and facilitators to children’s centres implementing the IPB.

Outcomes were ascertained 12 months post-intervention commencement in the IPB+ and IPB only arms and 12 months post-randomisation in the usual care arm, plus facilitation contact data which were collected at 1,3 and 8 months post commencement of the intervention in the IPB+ arm.

Outcomes were measured using a range of tools. Families completed baseline and 12 month follow-up questionnaires. Data from children’s centres included baseline and post-intervention manager-completed questionnaires, facilitation questionnaires and interviews with the IPB+ children’s centre staff at 1, 3 and 8 months and activity logs to record home safety activities. The two intervention arms also completed questionnaires and interviews to assess implementation fidelity at 12 months.[14] Baseline data from children’s centres were collected between August 2011 and January 2012 and follow-up data, including facilitation contact data were collected between June 2012 and June 2013. Parent baseline data were collected between January 2012 and May 2012 and follow-up data between May 2013 and September 2013. Follow-up data were analysed in 2014.

### Randomisation

Children’s centres were stratified by study site (4 strata) and randomly allocated within strata to one of the three study arms using permuted block randomisation, with fixed block sizes of 3; thus, in each of the four study sites, three children’s centres were allocated to each arm. The allocation schedule was produced by an independent statistician, using the Stata randomisation algorithm. Allocations were placed in sequentially numbered opaque envelopes (one set for each trial site). When a study site (stratum) had recruited its first three children’s centres, and each of those centres had recruited 30 families, the first three envelopes were opened by an administrator not otherwise involved in the trial and the allocation of those centres to study arm was revealed. The same process was followed after the recruitment of children’s centres 4–6 and 5–9 at each study site.

### Blinding

It was not possible to blind children’s centre staff, or researchers providing the intervention to treatment arm allocation, but parents were blinded to treatment arm allocation. Analyses were undertaken blind to treatment arm allocation.

### Sample size

The sample size was calculated for the primary outcome (a binary variable for whether or not the family had a plan for escaping from a house fire). The number of families and clusters required were obtained by calculating the sample size required for an individually randomised trial then applying the design effect derived from the intraclass correlation coefficient and the cluster size to account for clustering of families within children’s centres using published formulae for cluster randomised trials.[27] Eleven children’s centres per intervention arm (a total of 33 centres across the four study sites) were required to detect an absolute difference in the percentage of families with a plan for escaping from a house fire of 20% (equivalent to an odds ratio of 2.25) in either of the two intervention arms compared with the control arm. This assumed a prevalence of 42% for families in the control arm having a plan for escaping from a house fire, an intraclass correlation coefficient of 0.05,[28] outcomes being available on 20 families per children’s centre (giving a design effect of 1.95), 80% power and 5% significance level (2-sided). To allow for attrition, we increased the number of children’s centres to 36 and the average number of families recruited per centre to 30, giving a total of 1080 families across the four study sites (9 children’s centres and 270 families per study site).

### Statistical analysis

Baseline characteristics were summarised by treatment arm. Quantitative analyses were undertaken using a pre-specified analysis plan on an intention-to-treat basis using Stata versions 11 and 13.

#### Primary outcome

The primary outcome (a binary outcome of whether the family had a plan for escaping from a house fire) was analysed using random effects logistic regression to estimate odds ratios and 95% CIs, comparing families in the two intervention arms with the control arm, with children’s centre included as a random effect. The model included randomisation stratum (trial site) as a fixed effect and was adjusted for two children’s centre-level variables (lead agency (Local Authority, National Health Service or Voluntary sector) and Ofsted scores for overall effectiveness (Ofsted inspects and regulates services that care for children and young people and services providing education and skills for learners of all ages) and two family-level variables (plan for escaping from a house fire at baseline and Index of Multiple Deprivation (IMD) 2010 score based on home postcode).[16] The IMD is a small (400–1200 households) area-based measure of deprivation comprising seven domains (income, employment, health and disability, education skills and training, barriers to housing and services, living environment and crime).

As a secondary analysis, we tested for differential effects of interventions by deprivation by adding interaction terms to the model. We used one way analysis of variance to calculate the intraclass correlation coefficient.

#### Secondary outcomes

For secondary outcomes measured at family-level, we analysed binary outcomes using random effects logistic regression, the number of correct responses to fire safety knowledge questions using random effects ordinal regression and the bedtime safety routine score using random effects linear regression. Models were adjusted for the same baseline covariates included in the model for the primary outcome, as described above. Statistical analysis of secondary outcomes measured at children’s centre-level was not undertaken due to small numbers in some groups.

#### Sensitivity analyses

The main analyses for all outcomes were complete case analyses. For the primary outcome we undertook sensitivity analyses, (a) using multiple imputation to replace missing values and created 50 datasets which were combined using Rubin’s rules,[29] and, (b) assuming no change from baseline values in those lost to follow-up.

### Health economic evaluation

The cost-effectiveness analysis was conducted from a societal perspective and used the primary effectiveness endpoint of the trial and economic endpoint of the total cost of the intervention (expressed in 2012 UK£), with data analysed at family-level. Resource use and cost data were obtained from: i) activity logs (relating to implementation of intervention); ii) children’s centre follow-up questionnaires (detailing fire safety activities); iii) parent follow-up questionnaires (resources and costs related to fire safety sessions and home safety inspections). Study site and children’s centre-level costs were averaged equally across families within each study site and children’s centre, respectively, and combined with family-level costs to give a total cost per family. The primary outcome was cost per additional plan for escaping from a house fire estimated for the IPB only and IPB+ arms compared to the usual care arm.

We adopted a hierarchical modelling approach, allowing for clustering and adjusting for the baseline covariates included in the primary effectiveness analysis. This model extended recently developed methodology [30, 31] for cost-effectiveness analysis alongside cluster trials (see S1 Text for further details). This approach used Markov Chain Monte Carlo simulation to fit the non-standard statistical model using the WinBUGS software.[32] A summary of the base-case cost-effectiveness analysis is provided in Table 1.

**Table 1:** Summary of the base-case cost-effectiveness analysis.

Type of evaluation	Prospective cost-utility analysis alongside a cluster RCT
Time horizon	1-year
Perspective	Societal
Comparators	Usual care
	Injury prevention briefing
	Injury prevention briefing with facilitation
Cost categories	Children’s centre
	Fire and Rescue Service
	Other agencies including council
	Family
Base year for calculating costs/prices	2012 UK£
Analytic methods	Hierarchical model allowing for clustering and adjusting for the baseline covariates included in the primary effectiveness analysis
Outcome	Cost per additional fire escape plan

One of the children’s centres in the usual care group had extremely high costs and its impact on the results was assessed by excluding it in a sensitivity analysis. We checked robustness of findings to missing data by extending the imputation model described above for the primary outcome to include costs incurred by (a) parents, (b) fire and rescue services, (c) children’s centres and (d) other agencies. Since analysis was carried out using MCMC simulation it was not practical to perform 50 imputations (as done for effectiveness), instead 10 imputations were used.

### Qualitative analysis

Data from facilitation contacts were analysed using thematic analysis after categorisation into main sub-headings.[33] An analytical framework was developed and applied to implementation fidelity interviews using Framework Analysis,[34] supported by QSR NVivo 10.

### Changes to the protocol

There were six amendments made to the protocol following approval by the ethics committee. These were:

1. Specifying the trial was a cluster randomised controlled trial by adding the word “cluster” to the trial title in the protocol
2. Extending recruitment of children’s centres to those with more than 50% of their catchment population in the most 30% most deprived areas to ensure recruitment of sufficient children’s centres
3. Increasing the number of children’s centre managers interviewed in the intervention arms (from a sample of 18 to managers from all 24 centres) to ensure data on facilitators and barriers to implementation of the IPB were collected from all centres
4. Reducing data collection contacts between research team and the IPB only arm to a single contact at 12 months to reduce the likelihood that data collection contacts would act as prompts to action to implement guidance that would not usually occur when guidance documents are disseminated
5. The independent Trial Steering Committee (TSC) recommended modifying the data collection at facilitation contacts in the ‘IPB plus facilitation’ arm so that a two-stage approach was used, with a questionnaire sent prior to the interview to allow time for the data to be collected, rather than the questionnaire being completed at the interview.
6. The independent TSC advised using latent variable analysis on the baseline data to create a composite secondary outcome measure which measured a range of fire escape behaviours and to include this as an additional secondary outcome measure for the trial.[35] This was approved by the ethics committee on 7/2/13. This was prior to completion of follow up data collection and prior to any analysis of follow up data.

### Ethical and organisational review

Derbyshire Research Ethics Committee (11/EM/0011) and the University of the West of England, Bristol, Research Ethics Committee (HSC/11/06/61) provided ethical approval on 18^th^ March 2011 and 22^nd^ July 2011, respectively. Primary Care Trusts (PCTs) provided NHS organisational approval. Informed written consent was obtained from all participants involved.

## Results

### Recruitment and retention

**Fig 1:**
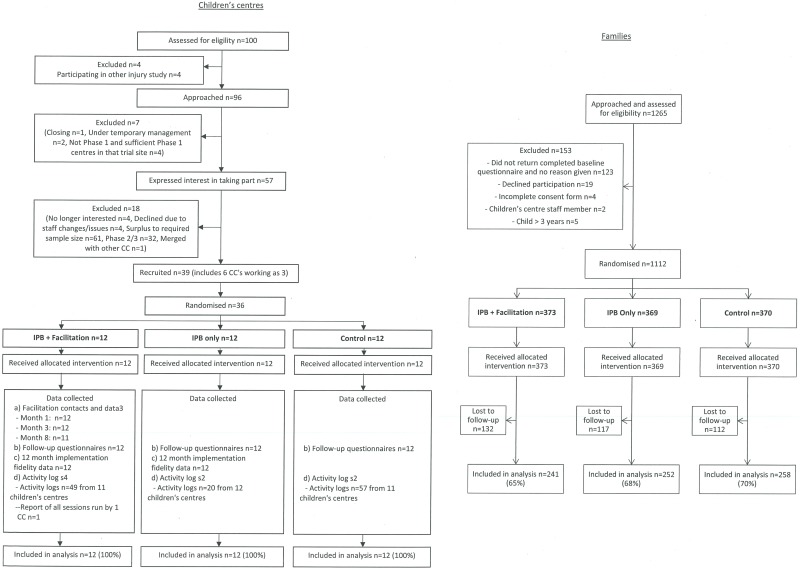
Flow of children’s centres and families through the trial.

Fig 1 shows the flow of children’s centres and families through the trial. Ninety-six children’s centres were approached, seven of which were excluded. Fifty-seven centres expressed interest in taking part, 18 of which were excluded. Thirty-nine centres were recruited, six of which were jointly operating as three children’s centres, giving a total of 36 centres. A total of 1265 families were approached, of whom 1112 were recruited. All children’s centres remained in the trial and 361 families (32%) were lost to follow-up. Loss to follow-up was similar across trial arms.

### Baseline characteristics

Table 2 shows the characteristics of children’s centres and families at baseline. Trial arms appear to be well balanced. Two fifths of families (42%) had an existing plan for escaping from a house fire with a similar proportion in each arm.

**Table 2:** Baseline characteristics of children’s centres and families.

	Trial Arm		
**Characteristics**	**Usual care n = 12**	**IPB only n = 12**	**IPB + facilitation n = 12**
**Children’s centres**			
Study centre:			
Nottingham	3	3	3
Newcastle	3	3	3
Norwich	3	3	3
Bristol	3	3	3
Lead agency:			
Local Authority	10	7	9
NHS	0	0	2
Voluntary sector	2	5	1
Phase 1 centre	10	11	11
Phase 2 centre	2	1	1
Number of children in catchment area: median (IQR)	754 (529, 999)	776 (565, 905)	854 (608, 1076)
Ofsted score for overall effectiveness:			[1]
Outstanding	3	2	4
Good/satisfactory	9	10	7
Children’s centre provides advice on:			
Smoke alarm use	12	10	12
How to make a plan for escaping from a house fire	9	[1] 9	9
Causes of house fires	12	11	12
Safe use and storage of cigarettes, lighters & matches	10	10	[1] 9
Bedtime routines to prevent fires	6	[1] 7	9
**Families**	**Usual care n = 370 (%)**	**IPB only n = 369 (%)**	**IPB + facilitation n = 373 (%)**
Study centre:			
Nottingham	89 (24.1)	98 (26.6)	91 (24.4)
Newcastle	86 (23.2)	88 (23.9)	87 (23.3)
Norwich	95 (25.7)	82 (22.2)	93 (24.9)
Bristol	100 (27.0)	101 (27.4)	102 (27.4)
Single adult household	[13] 61 (17.1)	[15] 72 (20.3)	[15] 59 (16.5)
Only 1 child in household	[12] 169 (47.2)	[13] 173 (48.6)	[16] 200 (56.0)
>1 family living in same household	29 (8.0)	37 (10.0)	37 (10.0)
Number of families with children aged:	[10]	[6]	[9]
under 1 year	163 (45.3)	143 (39.4)	178 (48.9)
1–2 years	197 (54.7)	220 (60.6)	186 (51.1)
Mother aged ≤20 years	[16] 17 (4.8)	[19] 17 (4.9)	[17] 20 (5.6)
Father aged ≤20 years	[57] 6 (1.9)	[67] 6 (2.0)	[59] 8 (2.6)
Family ethnicity: White British	[20] 337 (96.3)	[18] 323 (92.0)	[12] 348 (96.4)
English as first language	[5] 336 (92.1)	[5] 319 (87.6)	[2] 349 (93.1)
Rented accommodation	[13] 193 (54.1)	[6] 203 (55.9)	[6] 193 (52.6)
Deprivation mean (SD) (IMD of household)	[2] 31.0 (16.9)	[1] 34.7 (16.5)	[1] 29.6 (16.1)
No smokers in household	[11] 245 (68.3)	[9] 251 (69.7)	[10] 263 (72.5)
At least one person in household drinks ≥4 times/week	[10] 21 (5.8)	[8] 19 (5.3)	[12] 24 (6.7)
At least one person in household drinks ≥6 drinks on one occasion	[33] 208 (61.7)	[38] 173 (52.3)	[29] 211 (61.3)
Family have a plan for escaping from a house fire	[7] 159 (43.8)	[5] 153 (42.0)	[7] 149 (40.7)

[] = missing values

### Primary and secondary outcomes

There was no significant difference in the proportion of families with a plan for escaping from a house fire at 12 months between treatment arms (AOR IPB only vs. usual care: 0.93, 95%CI 0.58, 1.49; AOR IPB+ facilitation vs. usual care 1.41, 95%CI 0.91, 2.20) (Table 3; results for full model in S1 Table). There was no significant interaction between deprivation and the effect of the interventions (p = 0.86). The intraclass correlation coefficient for having a plan for escaping from a house fire at 12 months was 0.003 (95% CI 0.000, 0.027) (S2 Table).

**Table 3:** Primary and secondary outcomes at 12 month follow-up by treatment arm.

Outcome measures	Trial Arm			IPB only vs. usual care		IPB+ vs. usual care	
	Usual care n = 258 (%)	IPB only n = 252 (%)	IPB+ n = 241 (%)	Odds ratio (95% CI)	p value	Odds ratio (95% CI)	p value
**Primary outcome measure**^1^							
Family have a plan for escaping from a house fire:	[4]	[9]	[5]				
No	135 (53.2)	135 (55.6)	116 (49.2)	1.00		1.00	
Yes	119 (46.9)	108 (44.4)	120 (50.9)	0.93 (0.58, 1.49)	0.76	1.41 (0.91, 2.20)	0.13
**Secondary outcome measures**^2^							
Fire escape behaviours composite variable:							
Fewer fire escape behaviours	45 (17.4)	29 (11.5)	32 (13.3)	1.00		1.00	
More fire escape behaviours	213 (82.3)	223 (88.5)	209 (86.7)	2.56 (1.38, 4.76)	<0.01	1.78 (1.01, 3.15)	0.05
Smoke alarms fitted and working on every level:	[7]	[12]	[8]				
No	22 (8.8)	14 (5.8)	13 (5.6)	1.00		1.00	
Yes	229 (91.2)	226 (94.2)	220 (94.4)	1.61 (0.71, 3.66)	0.25	1.56 (0.71, 3.42)	0.27
Fire setting or match play by children:	[52]	[49]	[49]				
No	197 (95.6)	198 (97.5)	181 (84.3)	1.00		1.00	
Yes	9 (3.5)	5 (2.5)	11 (5.7)	0.27 (0.08, 0.94)	0.04	1.22 (0.43, 3.08)	0.77
Bedtime fire safety routine score (median(IQR)):^3^	[9] 8 (8, 9)	[16] 9 (8, 10)	[11] 8.5 (8, 9)	1.59 (1.09, 2.31)	0.02	1.22 (0.85, 1.76)	0.28
Took part in smoking cessation courses/support:	[60]	[43]	[43]				
No	5 (19.2)	8 (33.3)	5 (23.8)	1.00		1.00	
Yes	21 (80.8)	16 (66.7)	16 (76.2)	0.23 (0.04, 1.43)	0.12	0.61 (0.11, 3.40)	0.57
Number of correct responses to fire safety knowledge questions: ^3^							
0	78 (30.2)	81 (32.1)	70 (29.1)	1.10 (0.77, 1.57)	0.61	1.22 (0.86, 1.73)	0.26
1	93 (36.1)	86 (34.1)	76 (31.5)				
2	80 (31.0)	81 (32.1)	85 (35.3)				
3	7 (2.7)	4 (1.6)	10 (4.2)				
Satisfaction with home safety information provided by children’s centre:^4^	[57]	[55]	[53]				
Neither satisfied nor dissatisfied/fairly/very dissatisfied	16 (8.0)	22 (11.2)	23 (12.2)	1.00		1.00	
Very/fairly satisfied	31 (15.4)	46 (23.4)	73 (38.8)	1.08 (0.4, 2.8)	0.87	1.79 (0.7, 4.4)	0.20
No information received	154 (76.6)	129 (65.5)	92 (48.9)				

[] = missing values

^1^ Adjusted for study centre, lead agency of children’s centre (Local authority, NHS or Voluntary sector), OFSTED effectiveness score (Outstanding, Good, Satisfactory, Missing), fire escape plan at baseline (no/yes), IMD score of family (continuous). We have not adjusted for OFSTED capacity for sustained improvement as this variable had more missing data and where recorded the values are the same as for OFSTED overall effectiveness score.

^2^ Adjusted for the lead agency of the children’s centre, Ofsted report scores for overall effectiveness, baseline value of the secondary outcome measure, IMD. We have not adjusted for OFSTED capacity for sustained improvement as this variable had more missing data and where recorded the values are the same as for OFSTED overall effectiveness score.

^3^ Odds ratio for a one unit increase in the outcome measure.

^4^ Participants who had not received information were excluded from the analysis

Significantly more IPB only (AOR 2.56, 95%CI 1.38, 4.76) and IPB+ arm families (AOR 1.78, 95%CI 1.01–3.15) were in the “more behaviours for escaping from house fires” group than usual care arm families. Families in the IPB only arm were significantly less likely to report children playing with matches or lighters (AOR 0.27, 95%CI 0.08, 0.94) and reported significantly more bedtime fire safety routines than usual care arm families (AOR for a one unit increase in number of bedtime fire safety routines AOR 1.59, 95%CI 1.09, 2.31).

Table 4 shows a significantly higher proportion of IPB+ arm families reported receiving advice about each of the five key IPB messages and attended fire safety sessions on each of the five key IPB messages than control arm families. A significantly higher proportion of IPB only arm families attended fire safety sessions on three of the five key IPB messages than control arm families. A significantly higher proportion of families in both intervention arms reported attending a fire safety session than control arm families. Only a small proportion of families attended two or more fire safety sessions (usual care: 3.9%, IPB only: 11.9%, IPB+ 19.3%). There were no significant differences in other secondary outcome measures. Table 5 shows the fire safety activities reported by children’s centres. Numbers were too small for statistical analysis but the findings are consistent with family-reported fire safety activities.

**Table 4:** Receipt of fire safety advice and other fire safety promotion by families at follow-up, by treatment arm.

Receipt of fire safety advice and promotion	Trial Arm			IPB only vs. usual care		IPB+ vs. usual care	
	Usual care n = 258 (%)	IPB only n = 252 (%)	IPB+ n = 241 (%)	Odds ratio (95% CI)^1^	p value	Odds ratio (95% CI)^1^	p value
Received advice on the five key IPB messages:							
i. Smoke alarms^2^	[54]	[52]	[54]				
No	155 (75.6)	132 (66.0)	107 (57.2)	1.00		1.00	
Yes	49 (24.0)	68 (34.0)	80 (42.8)	1.36 (0.82, 2.26)	0.23	2.27 (1.40, 3.67)	p<0.01
ii. Matches^2^	[56]	[57]	[58]				
No	177 (87.6)	167 (85.6)	133 (72.7)	1.00		1.00	
Yes	25 (12.4)	28 (14.4)	50 (27.3)	1.05 (0.54, 2.04)	0.89	2.74 (1.51, 4.96)	p<0.01
iii. Fire escape plans	[55]	[58]	[57]				
No	175 (86.2)	168 (86.6)	133 (72.3)	1.00		1.00	
Yes	28 (13.8)	26 (13.4)	51 (27.7)	0.79 (0.40, 1.55)	0.50	2.38 (1.35, 4.21)	p<0.01
iv. Bedtime safety routines	[54]	[56]	[56]				
No	183 (89.7)	173 (88.3)	147 (79.5)	1.00		1.00	
Yes	21 (10.3)	23 (11.7)	38 (20.5)	0.89 (0.44, 1.82)	0.76	2.21 (1.18, 4.12)	p<0.01
v. Causes of fires	[57]	[56]	[57]				
No	169 (84.1)	149 (76.0)	113 (61.4)	1.00		1.00	
Yes	32 (15.9)	47 (24.0)	71 (38.6)	1.50 (0.85, 2.65)	0.17	3.35 (1.98, 5.68)	p<0.01
Number of key safety messages had advice on:	[53]	[52]	[52]				
2 or less	180 (87.8)	170 (85.0)	132 (69.8)	1.00		1.00	
3–5	25 (12.2)	30 (15.0)	57 (30.2)	1.09 (0.57, 2.10)	0.80	3.06 (1.72, 5.43)	p<0.01
Attended a fire safety session in the last year:	[53]	[50]	[49]				
No	197 (96.1)	178 (88.1)	155 (80.7)	1.00		1.00	
Attended 1 or more	8 (3.9)	24 (11.9)	37 (19.3)	3.20 (1.27, 8.06)	0.01	7.07 (3.05, 16.38)	p<0.01
Attended a fire safety session at children’s centre:	[53]	[50]	[49]				
No	197 (96.1)	185 (91.6)	163 (84.9)	1.00		1.00	
Attended 1 or more	8 (3.9)	17 (8.4)	29 (15.1)	2.18 (0.85, 5.63)	0.11	5.14 (2.20, 12.03)	p<0.01
Attended fire safety session about each of the five key messages in the IPB in the last year:*	[53]	[50]	[49]				
i. Smoke alarms							
No	198 (96.6)	180 (89.1)	158 (82.3)	1.00		1.00	
Yes	7 (3.4)	22 (10.9)	34 (17.7)	3.34 (1.30, 8.58)	0.01	6.71 (2.80, 16.04)	p<0.01
ii. Matches							
No	201 (98.1)	189 (93.6)	169 (88.0)	1.00		1.00	
Yes	4 (2.0)	13 (6.4)	23 (12.0)	2.80 (0.85, 9.29)	0.09	6.78 (2.24, 20.55)	p<0.01
iii. Fire escape plans							
No	201 (98.1)	188 (93.1)	162 (84.4)	1.00		1.00	
Yes	4 (2.0)	13 (6.9)	30 (15.6)	3.48 (1.06, 11.44)	0.04	9.88 (3.31, 29.43)	p<0.01
iv. Bedtime safety routines							
No	202 (98.5)	189 (93.6)	172 (89.6)	1.00		1.00	
Yes	3 (1.5)	13 (6.4)	20 (10.4)	3.93 (1.04, 14.93)	0.04	7.83 (2.23, 27.55)	p<0.01
v. Causes of fires							
No	198 (96.6)	184 (91.1)	162 (84.4)	1.00		1.00	
Yes	7 (3.1)	18 (8.9)	30 (15.6)	0.56 (0.0, 11.9)	0.06	5.52 (2.29, 13.30)	p<0.01

[] = missing

* Some families attended more than 1 session.

^1^ Adjusted for study centre, lead agency of children’s centre (Local authority, NHS or Voluntary sector), OFSTED effectiveness score (Outstanding, Good, Satisfactory, Missing), baseline value of the secondary outcome measure, IMD score of family (continuous). We have not adjusted for OFSTED capacity for sustained improvement as this variable had more missing data and where recorded the values are the same as for OFSTED overall effectiveness score.

^2^ IMD quintiles used because of non-linear association with the outcome.

**Table 5:** Fire safety activities reported by children’s centres at follow-up, by treatment arm.

Secondary outcome measures	Trial arm		
	Usual care	IPB only	IPB+ facilitation
	n = 12	n = 12	n = 12
Advice provided on:			
Smoke alarms		[1]	
No advice / Don’t know	1	0	1
Yes	11	11	11
How to make a fire escape plan	[1]		
No advice / Don’t know	3	3	0
Yes	8	9	12
Causes of house fires (cooking safety, electrical safety, handling hot irons safely)			
No advice / Don’t know	1	0	0
Yes	11	12	12
Child behaviour and fire prevention (safe use and storage of cigarettes, lighters and matches)			
No advice / Don’t know	4	11	0
Yes	8	1	12
Bedtime routines to prevent fires	[1]		[1]
No advice / Don’t know	6	4	0
Yes	5	8	11
Children’s centre provided fire safety sessions	[1]		
No	6	5	1
Yes-	5	7	11
Mean number of sessions (min to max)*	1.2 (1 to 2)	2.1 (1 to 4)	3.1 (1 to 7)
Mean session length in minutes (min to max)	116 (90 to 120)	90 (30 to 120)	89 (30 to 130)
FRS attended to help provide any sessions	[1]		[1]
No	8	6	4
yes	3	6	7

[] missing values.

*assumes those who said they ran a session but didn’t answer question on number of sessions, ran only one session.

FRS = fire and rescue service

### Sensitivity analyses

AORs from the multiple imputation analysis for the primary outcome were similar to those from the complete case analysis (IPB only: AOR 0.92, 95%CI 0.58, 1.46; IPB+: AOR 1.40, 95%CI 0.89, 2.21) and from the analysis assuming no change from baseline (IPB only: AOR 0.95, 95%CI 0.60, 1.51; IPB+: AOR 1.39, 95%CI 0.91, 2.12).

### Health economics

Details about derivation of costings are presented in Tables 6–9. The cost of developing the IPB (£15,860) was excluded from the cost-effectiveness analysis as this fixed, one-off cost would not be encountered again if this intervention was implemented in practice.

**Table 6:** Sources of unit cost data (UK£ 2012).

	Value	Source
**Parent costs**		
Time costs	£45.70/hour	Department for Transport. TAG UNIT 3.5.6 Values of Time and Vehicle Operating Costs Transport Analysis Guidance (TAG). Available from www.dft.gov.uk/webtag [Accessed October 2012].
Travel costs by car	£0.18/km	Department for Transport. TAG UNIT 3.5.6 Values of Time and Vehicle Operating Costs Transport Analysis Guidance (TAG). Available from www.dft.gov.uk/webtag [Accessed October 2012].
**IPB implementation costs**		
Researcher’s time	£19.04/hour	University of Nottingham pay scale
Administrator’s time	£11.24/hour	University of Nottingham pay scale
**Children’s centre, Fire and Rescue Service and other agency costs**		
FRS staff time	£36.00/hour	Personal communication, Adam Shaw, Cheshire Fire and Rescue Service, 20 September 2012,
Children’s centre staff’s time	£18.00/hour	Personal Social Services Research Unit. Unit costs of health and social care 2012, 2012. (assumed same as home care worker)
Home inspection	£15.33	Based on 40 minute visit by children’s centre, FRS or other agency (as in decision models)

FRS = fire and rescue service

**Table 7:** Costs of providing the IPB, training and facilitation (UK £2012).

Arm	Study centre	Number of families randomised	Number of children’s centres	IPB printing & distribution	IPB training session	IPB facilitation	Total per study centre	Total per children’s centre	Total per family randomised
Usual care	Bristol	100	3	£0.00	£0.00	£0.00	£0.00	£0.00	£0.00
	Newcastle	86	3	£0.00	£0.00	£0.00	£0.00	£0.00	£0.00
	Norwich	95	3	£0.00	£0.00	£0.00	£0.00	£0.00	£0.00
	Nottingham	89	3	£0.00	£0.00	£0.00	£0.00	£0.00	£0.00
IPB only	Bristol	101	3	£152.50	£0.00	£0.00	£152.50	£50.83	£1.51
	Newcastle	88	3	£152.50	£0.00	£0.00	£152.50	£50.83	£1.73
	Norwich	82	3	£152.50	£0.00	£0.00	£152.50	£50.83	£1.86
	Nottingham	98	3	£152.50	£0.00	£0.00	£152.50	£50.83	£1.56
IPB +	Bristol	102	3	£152.50	£1,328.95	£327.84	£1,809.29	£603.10	£17.74
	Newcastle	87	3	£152.50	£1,408.84	£220.57	£1,781.91	£593.97	£20.48
	Norwich	93	3	£152.50	£1,488.74	£127.90	£1,769.14	£589.71	£19.02
	Nottingham	91	3	£152.50	£1,568.63	£84.74	£1,805.87	£601.96	£19.84

**Table 8:** Other intervention costs expressed per cluster (i.e. children’s centre) and per family.

	Usual care - Mean (Min to Max)		IPB only - Mean (Min to Mix)		IPB + facilitation - Mean (Min to Max)	
	per cluster	per family	per cluster	per family	per cluster	per family
	9 clusters	151 families	9 clusters	140 families	9 clusters	123 families
**Fire safety sessions**						
Children’s centre costs	£421.00 (0.00 to 1800*)	£13.72 (0.00 to 62.07)	£63.00 (0.00 to 198.00)	£2.06 (0.00 to 7.07)	£222.00 (0.00 to 900.00)	£7.63 (0.00 to 32.14)
Fire & Rescue service costs	£74.38 (0.00 to 378.00)	£2.57 (0.00 to 13.03)	£68.66 (0.00 to 288.00)	£2.20 (0.00 to 9.60)	£136.49 (0.00 to 372.00)	£4.38 (0.00 to 11.63)
Parent costs to attend sessions	£2.59 (0.00 to 22.85)	£0.15 (0.00 to 22.85)	£9.15 (0.00 to 22.85)	£0.59 (0.00 to 17.14)	£14.83 (0.00 to 62.32)	£1.09 (0.00 to 35.18)
**Home safety inspections**						
Children’s centre costs	£3.41 (0.00 to 15.33)	£0.20 (0.00 to 15.33)	£6.81 (0.00 to 30.66)	£0.44 (0.00 to 15.33)	£8.52 (0.00 to 30.66)	£0.62 (0.00 to 15.33)
Fire & Rescue Service costs	£13.63 (0.00 to 30.66)	£0.76 (0.00 to 15.33)	£13.63 (0.00 to 30.66)	£1.07 (0.00 to 15.33)	£22.14 (0.00 to 76.65)	£1.62 (0.00 to 15.33)
Other agencies	£10.22 (0.00 to 45.99)	£0.61 (0.00 to 15.33)	£22.14 (0.00 to 45.99)	£1.42 (0.00 to 45.99)	£15.33 (0 to 45.99)	£1.12 (0.00 to 15.33)

Complete data on costs available for 9 clusters per arm. The number of families represents the number of families with complete data within the 9 clusters per arm.

*Possible outlier (reported 20 children’s centre staff + 5 Fire & Rescue Service staff providing a “fun day” (cluster 6, usual care arm))—when this cluster is removed the mean is reduced to £254.25 with a maximum per cluster of £792.00.

**Table 9:** Total intervention costs expressed per cluster (i.e. children’s centre) and per family.

	Usual care - Mean (Min to Max)		IPB only - Mean (Min to Mix)		IPB + facilitation - Mean (Min to Max)	
	per cluster	*per family*	per cluster	per family	per cluster	per family
	9 clusters	151 families	9 clusters	140 families	9 clusters	123 families
IPB provision, training and facilitation plus other intervention costs	£303.01 (30.66 to 1367.67)	£18.06 (0.00 to 90.43)	£143.68 (62.51 to 240.22)	£9.24 (1.51 to 46.79)	£507.81 (290.26 to 859.92)	£37.16 (21.18 to 79.20)
Other intervention costs only	£303.01 (30.66 to 1367.67)	£18.06 (0 to 90.43)	£117.87 (45.89 to 210.58)	£7.58 (0.00 to 45.06)	£224.97 (110.66 to 497.62)	£16.46 (2.70 to 51.33)

**Fig 2:**
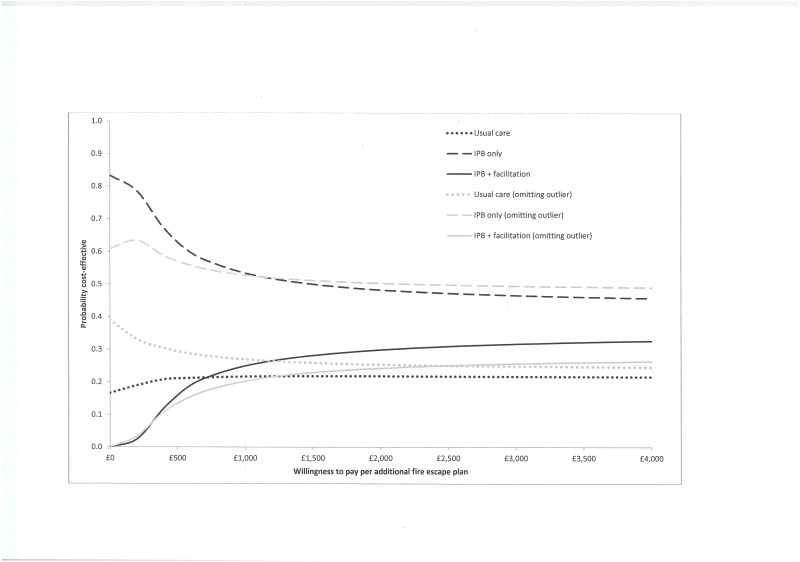
Cost-effectiveness acceptability curves for the complete case analysis and without the outlier children’s centre.

Table 10 presents the results of the cost-effectiveness analysis comparing IPB only and IPB+ to usual care arms. The complete-case analysis shows the IPB only is both less costly and more effective than usual care, whereas the IPB+ is more costly and more effective than usual care. As the inverse of the difference in probabilities of having a plan for escaping from a house fire between two treatment arms is equal to the Number Needed to Treat (NNT), the cost-effectiveness ratios can be interpreted as the cost per additional plan for escaping from a house fire under the intervention. The cost-effectiveness acceptability curves (Fig 2) show the IPB only has the highest probability of being cost-effective at a decision maker’s willingness to pay between £0 and £4,000 (US$ 6061) per additional plan for escaping from a house fire.

**Table 10:** Cost-effectiveness analysis results for complete case and sensitivity analyses.

	Usual care	IPB only	IPB + facilitation	IPB only vs. Usual care	IPB + facilitation vs. Usual care
**Complete case analysis**					
**Number of families**	151	140	123		
**Number of children’s centres**	9	9	9		
Mean cost per family (95% Credible interval (CrI))	£21.15 (3.95 to 38.31)	£12.65 (4.66 to 20.03)	£41.41 (31.58 to 52.41)	-£8.49	£20.26
Proportion with Fire escape plan (95% CrI)	0.48 (0.35 to 0.56)	0.49 (0.38 to 0.58)	0.48 (0.37 to 0.58)	0.03	0.02
Incremental cost effectiveness ratio				-£275.31	£1007.96
Probability cost effective @ £200 (US$ 306) per additional plan for escaping from a house fire	0.19	0.78	0.02		
Probability cost effective @ £1000 (US$ 1531) per additional plan for escaping from a house fire	0.22	0.53	0.25		
**Sensitivity analysis omitting outlying cluster**					
Number of families	151	140	123		
Number of children’s centres	9	9	9		
Mean cost per family (95% CrI)	£14.99 (6.16 to 24.11)	£13.26 (4.52 to 22.24)	£39.97 (31.25 to 48.41)	-£1.74	£24.98
Proportion with plan for escaping from a house fire (95% CrI)	0.47 (0.34 to 0.61)	0.50 (0.37 to 0.64)	0.48 (0.34 to 0.62)	0.03	0.01
Incremental cost effectiveness ratio				-£53.01	£3778.55
Probability cost effective @ £200 (US$ 306) per additional plan for escaping from a house fire	0.33	0.63	0.03		
Probability cost effective @ £1000 (US$ 1531) per additional plan for escaping from a house fire	0.27	0.53	0.20		
**Sensitivity analysis imputing for missing values**					
Number of families	370	369	373		
Number of children’s centres	12	12	12		
Mean cost per family (95% CrI)	£19.21 (14.64 to 23.77)	£10.60 (6.48 to 14.73)	£43.01 (38.71 to 47.30)	-£8.60	£23.80
Proportion with plan for escaping from a house fire (95% CrI)	0.44 (0.37 to 0.52)	0.44 (0.37 to 0.52)	0.58 (0.50 to 0.65)	-0.00	0.13
Incremental cost effectiveness ratio				£6447.53	£177.61
Probability cost effective @ £200 (US$ 306) per additional plan for escaping from a house fire	0.02	0.60	0.24		
Probability cost effective @ £1000 (US$ 1531) per additional plan for escaping from a house fire	0.00	0.02	0.96		

A sensitivity analysis, excluding the children’s centre with the potentially outlying cost, resulted in slight changes as reported in Table 10 and Fig 2. Analysis of imputed data, at values of a decision maker’s willingness to pay above £400 (US$ 606) per additional plan for escaping from a house fire, showed the IPB+ had the highest probability of being cost-effective (Table 10) reaching a probability of nearly 1 at £1000 (US$ 1515) per additional fire-escape plan. However, results should be interpreted with caution due to the large proportion of missing data imputed (ranging from just under 50% (families’ costs) to nearly 60% (children’s centre costs)).

### Qualitative analysis

The qualitative data have been reported previously.[14] However, the results provided clear indications of factors, e.g. organisational change, time and resources which moderated all children’s centres’ ability to implement the IPB. More specific factors which adversely affected implementation of the IPB by some children’s centres were also identified, e.g. staff training and continuity.

## Discussion

### Main findings

This three-arm cluster randomised controlled trial examined the effects of a complex intervention on fire safety behaviours in the home. We found that families in both intervention arms reported significantly more behaviours for escaping from house fires. Families in both intervention arms were significantly more likely to report attending a fire safety session than usual care arm families. However, in terms of the primary outcome measure families in either intervention arm were not significantly more likely to report having a plan for escaping from a house fire than usual care arm families. Economic analysis showed the IPB only intervention was both less costly and more effective than usual care, whereas the IPB + facilitation was more costly but also more effective than usual care.

### Strengths and limitations

Our trial used a theoretically-based intervention. It measured a wide range of outcome and process measures and used quantitative and qualitative approaches. This provided a good understanding of what the intervention comprised of and how it may have achieved its impact. Recruitment exceeded our required sample size; the attrition rate was consistent with the sample size calculation and was similar across treatment arms. The majority of participants were from the most disadvantaged areas hence our intervention was delivered to families at higher risk of injury.

In terms of limitations, few participants came from a black or ethnic minority group or had English as a second language. Outcome measures were self-reported and although we were able to blind parents to treatment arm allocation, children’s centre staff could not be blinded; hence this may have influenced reporting of some secondary outcome measures by children’s centres. Further work is required to explore the possibility that the intervention increased families’ understanding and hence their reporting of plans for escaping from house fires. The trial had three arms and multiple secondary outcome measures leading to multiple significance testing, although we did pre-specify a single primary outcome measure. We did not adjust significance levels to account for the three arm design or for having multiple secondary outcomes, since there is no consensus on this and it has been stated that formal adjustments for multiplicity usually complicate rather than enlighten.[36, 37] The significant results for secondary outcomes should however be interpreted with caution. Finally, some families did not receive the intervention and it is possible, therefore, that greater implementation may have achieved greater behavioural change. A recent national evaluation of children’s centres in the UK found most children’s centre services were used by families for less than one year.[38] Under these conditions it is difficult to achieve high levels of penetration of interventions.

### Comparisons with existing research

The baseline prevalence of plans for escaping from house fires in our study was similar to that in the US (52%), suggesting similar scope for improvement as in the UK. We were unable to find any published evaluations of injury prevention interventions delivered in children’s centres in the UK with which to compare our findings. Sure Start Local Programmes (SSLPs) were the forerunners to children’s centres. The national evaluation of SSLPs compared outcomes in SSLP families with those in the Millennium Cohort Study living in similarly disadvantaged areas without SSLPs.[39] At the age of three years, children in SSLP areas had a significantly lower unintentional injury rate than those in the non-SSLP areas; this difference was not maintained by the time children reached five years of age. Consistent with our findings, this suggests such services can impact on home safety outcomes for families in disadvantaged areas.[40] There have been few evaluations of Head Start programmes in the US with injury prevention as the focus even though children in the programmes are known to be at risk of injury.[41, 42]

Children’s centres in the UK have been evaluated in a multi-component six year study (The Evaluation of Children’s Centres in England (ECCE)). A survey of 5,700 parents participating in ECCE [38] suggested few (8%) had received home safety advice from children’s centres. The challenges of delivering evidence-based programmes within children’s centres were explored in questionnaires and interviews with staff in 121 children’s centres.[43] Widespread use of evidence-based programmes, particularly parenting programmes, was found. Education and training are important, not only for parents, but also for those who care for children and influence children.[2] By introducing the IPB to children’s centre staff both the staff and parents had exposure to evidence-based messages. However, as the ECCE study found, some children’s centre staff “gave equal weight to research evidence and personal experience” and only a small number of families were reached by the best evidenced programmes.[43] Similarly, we found fewer than 50% of families received each key safety message and fewer than 20% attended fire safety sessions.

## Conclusion and recommendations

This research has demonstrated that children’s centres can effectively deliver evidence-based injury prevention to families with young children who live in disadvantaged communities and achieve changes in some home safety behaviours. The economic analysis suggested the IPB only appeared to be most cost-effective, but this should be interpreted with caution as the results were sensitive to missing data.

Choosing outcome measures for fire prevention programmes is difficult. Fire-related injuries are uncommon events, and an extremely large trial would be required to evaluate the impact of an educational intervention on reducing fire-related injuries. There is evidence that some fire safety behaviours do reduce the risk of fire-related injuries and deaths, such as smoke alarms.[5, 6] However, smoke alarm ownership is very common in the UK (more than 90% of families in our trial reported a fitted and working smoke alarm on every level of their home at baseline), so demonstrating increases in functional ownership again requires large sample sizes. Furthermore, those who don’t already have smoke alarms may be particularly resistant to change, making only small effect sizes feasible to detect. We used fire escape plans as the primary outcome measure for our trial and also measured component elements of fire escape plans as a secondary outcome measure. Our finding of significant increases in the component elements of a plan but not in fire escape plans per se, suggests further work is needed to develop a valid and reliable measure of fire escape plans. Research demonstrating a reduction in risk of fire-related injury associated with having a fire escape plan would be helpful, as would larger trials allowing detection of smaller, but clinically important differences.

The mode of intervention, through children’s centre staff rather than directly to families, is typical of many health promotion programmes in practice. Our trial suggests less than full implementation of the IPB, hence future studies should consider additional or alternative implementation strategies.

### What is already known on this subject?

Little is known about effective measures to reduce the risk of fire-related death and injury in children other than provision of smoke alarms.

Children’s centres in the UK and Head Start in the US both have injury prevention as one of their priorities.

Educational interventions can increase the prevalence of plans for escaping from house fires although the effectiveness of children’s centres in delivering such packages is unknown.

### What this study adds?

An Injury Prevention Briefing (an evidence-based educational package delivered by children’s centres) can improve some aspects of families’ behaviours for escaping from house fires, e.g., making sure exits are clear.

The IPB only intervention was less costly and more effective than usual care, whereas the IPB + facilitation was more costly but also more effective than usual care. These results open the way for the development and evaluations of Injury Prevention Briefings for other types of injury.

### Trial registration

Trial registration: ClinicalTrials.gov identifier: NCT01452191. Date of registration: 10/10/2011. ISRCTN trial identifier: ISRCTN65067450. Date of assignation: 06/12/2012. The first children’s centre was recruited to the trial on 3/8/2011. The first parent was recruited to the trial on 5/1/2012. Trial registration occurred after recruitment of some children’s centres as the original protocol approved by the ethics committee did not include the word “cluster” in the title and we wished to register the trial with this specified in the title. This required a protocol amendment and ethical approval for the amendment.

## Supporting information

S1 Table Full model for primary outcome (family have a plan for escaping from a house fire) at 12 months follow-up. (DOCX)

S2 Table Analysis of variance table for primary outcome (family have a plan for escaping from a house fire) at 12 months follow-up according to clustering by children’s centre. (DOCX)

S1 Text Technical appendix. (DOCX)

S2 Text KCS Non-IMP interventional trial protocol V1. (DOCX)

S3 Text PLoS ONE CONSORT extension for cluster trials checklist. (DOCX)
