# Supplementary material for: Evaluating implementation of a fire-prevention injury prevention briefing in children's centres: Cluster randomised controlled trial
Source: PLoS One. 2017 Mar 24;12(3):e0172584. doi: 10.1371/journal.pone.0172584 (PMC5365108; doi:10.1371/journal.pone.0172584)
Supplement: S1 Table — (DOCX) [file pone.0172584.s001.docx]

**S1 Table** Full model for primary outcome (family have a plan for escaping from a house fire) at 12 months follow-up

|  | Odds ratio | (95% CI) | P value |
| --- | --- | --- | --- |
| *Study arm:* |  |  |  |
| Usual care | 1.00 |  |  |
| IPB only | 0.93 | (0.58, 1.49) | 0.76 |
| IPB+ | 1.41 | (0.91, 2.20) | 0.13 |
| *Study centre:* |  |  |  |
| Nottingham | 1.00 |  |  |
| Newcastle | 1.08 | (0.60, 1.95) | 0.80 |
| Norwich | 0.86 | (0.50, 1.48) | 0.59 |
| Bristol | 0.61 | (0.36, 1.04) | 0.07 |
| *Lead agency of children’s centre:* | |  |  |
| Local authority | 1.00 |  |  |
| NHS or Voluntary sector | 0.78 | (0.51, 1.20) | 0.27 |
| *OFSTED effectiveness score* |  |  |  |
| Outstanding | 1.00 |  |  |
| Good | 0.78 | (0.51, 1.19) | 0.25 |
| Satisfactory | 0.65 | (0.34, 1.28) | 0.21 |
| Missing | 0.98 | (0.31, 3.08) | 0.97 |
| Had fire escape plan at baseline | 9.06 | (6.37, 12.90) | <0.01 |
| IMD score of family (per unit) | 1.00 | (0.99, 1.02) | 0.40 |
