# Supplementary material for: Evaluating implementation of a fire-prevention injury prevention briefing in children's centres: Cluster randomised controlled trial
Source: PLoS One. 2017 Mar 24;12(3):e0172584. doi: 10.1371/journal.pone.0172584 (PMC5365108; doi:10.1371/journal.pone.0172584)
Supplement: S2 Table — (DOCX) [file pone.0172584.s002.docx]

**S2 Table** Analysis of variance table for primary outcome (family have a plan for escaping from a house fire) at 12 months follow-up according to clustering by children’s centre.

| Source | SS | df | MS | F | P value |
| --- | --- | --- | --- | --- | --- |
|  |  |  |  |  |  |
| Between clusters | 9.17924 | 35 | 0.262264 | 1.05 | 0.387 |
| Within clusters | 173.552 | 697 | 0.248999 |  |  |
|  |  |  |  |  |  |
| Total | 182.7312 | 732 | 0.249633 |  |  |

Intraclass correlation coefficient= 0.00261, 95% CI (0.00000 to 0.02737).
