# Supplementary material for: Evaluating implementation of a fire-prevention injury prevention briefing in children's centres: Cluster randomised controlled trial
Source: PLoS One. 2017 Mar 24;12(3):e0172584. doi: 10.1371/journal.pone.0172584 (PMC5365108; doi:10.1371/journal.pone.0172584)
Supplement: S1 Text — (DOCX) [file pone.0172584.s003.docx]

**TECHNICAL APPENDIX**

**Statistical model for the cost-effectiveness analysis**

The cost-effectiveness analysis was carried out with family as unit of analysis and, as for the clinical analysis of the trial, the clustered nature of the trial design (i.e. randomisation was at the Children Centre) needed to be accounted for([1](#_ENREF_1)). Additionally, the clinical and cost endpoints may themselves be correlated within families, as well as within Children Centre clusters, and thus the analysis needed to simultaneously allow for this. We adopted a hierarchical modelling approach here([2](#_ENREF_2)) which extended the hierarchical model used to analyse the effectiveness data in the primary clinical analysis. The effectiveness outcome (family have fire escape plan) in our application is dichotomous and therefore we had to adopt a multivariate methods for analysing a mixture of continuous and dichotomous outcomes([3](#_ENREF_3)) allowing for clustering([4](#_ENREF_4)). The model factorised the likelihood for costs and effects into the product of a marginal and conditional likelihood (continuous for costs and logistic for effects)([3](#_ENREF_3)). Within this model specification total cost at the family level was model using a Gamma distribution to allow for heavily (right) skewed data([5](#_ENREF_5)). Note to fit a Gamma distribution £1 had to be added to each family cost due the presence of zero costs in the dataset.

Algebraically, the model specified as follows:

$$e_{ijk}\sim Bernulli(p_{ijk})$$

$$c_{ijk}\sim Gamma\left( \eta_{k},\lambda_{ijk} \right)$$

$$logit\left( p_{ijk} \right)=\mu_{j}^{e}+\beta_{t}^{e}\times t_{i}$$

$$\lambda_{ijk}=\frac{\eta_{k}}{\varphi_{ijk}}$$

$$\varphi_{ijk}=\mu_{j}^{c}+\beta_{j}\times\left( e_{ijk}-p_{ijk} \right)+\boldsymbol{\beta}_{\boldsymbol{t}}^{\boldsymbol{c}}\times\boldsymbol{t}_{\boldsymbol{i}}$$

$\mu_{j}^{e}\sim Normal(\theta_{j}^{e},{\tau.e}^{2}$)

$\mu_{j}^{c}\sim Normal(\theta_{j}^{c},{\tau.c}^{2}$)

$$\theta_{j}^{e}=\mu_{k}^{e.clus}+\beta_{t}^{e.clus}\times t_{i}$$

$$\theta_{j}^{c}=\mu_{k}^{c.clus}+\beta_{k}^{c.clus}\times\left( \mu_{j}^{e}-\theta_{j}^{e} \right)+\boldsymbol{\beta}_{\boldsymbol{t}}^{\boldsymbol{c.clus}}\times\boldsymbol{t}_{\boldsymbol{j}}$$

Where $e_{ijk}$ and $c_{ijk}$ are the effects (*e*=0 no fire escape plan; *e*=1 fire escape plan exists) and costs in the *i*th family of the *j*th cluster allocated to the *k*th intervention group (*k*=1 Usual care, *k*=2 IPB only, *k*=3 IPB + facilitation). $p_{ijk}$ is the underlying probability of a fire escape plan at the family level, $\eta_{k}$ is the shape parameter of the Gamma distribution and is intervention group specific, and $\lambda_{ijk}$ is the rate parameter of the Gamma distribution at the family level. $\varphi_{ijk}$ is the underlying mean of the costs (and is a function of$\lambda_{ijk}$ and $\eta_{k}$) at the family level. $\mu_{j}^{e}$ is the underlying mean effect on the logit scale for the *j*th cluster, and $\mu_{j}^{c}$ is the intercept of the linear predictor for cost for the *k*th cluster. $\beta_{j}$ is the regression coefficient which links the cost and effect equations at the family level and is treatment arm specific and $\boldsymbol{\beta}_{\boldsymbol{t}}^{\boldsymbol{e}}$ and $\boldsymbol{\beta}_{\boldsymbol{t}}^{\boldsymbol{c}}$ are the regression coefficients for the *t* baseline family level covariates included in the model (i.e. the same covariates as included in the clinical effectiveness analysis). $\theta_{j}^{e}$ (defined further down as $\mu_{k}^{e.clus}$ ) and $\theta_{j}^{c}$ are the underlying cluster specific means, within a intervention group, are assumed to be exchangeable and Normally distributed with variance ${\tau.e}^{2}$ and ${\tau.c}^{2}$ respectively. $\mu_{k}^{c.clus}$ is the intercept of the linear predictor for cost for the underlying mean effect and $\beta_{k}^{c.clus}$ is the regression coefficient which links the cost and effect equations at the cluster level. Finally $\boldsymbol{\beta}_{\boldsymbol{t}}^{\boldsymbol{e.clus}}$ and $\boldsymbol{\beta}_{\boldsymbol{t}}^{\boldsymbol{c.clus}}$ are the regression coefficients for the *t* baseline cluster level covariates included in the model (i.e. the same covariates as included in the clinical effectiveness analysis). Note that following preliminary runs of the model, the coefficient linking the cost and effect equations at the cluster level was omitted because it did not significantly improve the fit of the model.

The cost-effectiveness model was fitted using the WinBUGS([6](#_ENREF_6)) software which allows great flexibility in model specification and estimates model parameters using Markov Chain Monte Carlo (MCMC) methods. WinBUGS uses the Bayesian statistical approach to inference, and as such requires prior distributions be placed on all unknown model parameters. For all parameters, vague prior distributions were specified, allowing the data to dominate the analysis. Following preliminary analyses checking convergence and autocorrelation between consecutive samples, the model was “burnt in” for 20,000 iterations followed by a further 180,000 iterations on which parameter estimation and inference was based. Convergence of the MCMC sampler was assessed using the Gelman Rubin diagnostic by running multiple chains with different starting values([7](#_ENREF_7)).

1. Gomes M, Ng E, Grieve R, Nixon R, Carpenter J, Thompson SG. Developing appropriate methods for cost-effectiveness analysis of cluster randomized trials. Medical Decision Making. 2012;32(2):350-61. Epub 2011 Oct 19.

2. Grieve R, Nixon R, Thompson S. Bayesian hierarchical models for cost-effectiveness analyses that use data from cluster randomized trials. Medical Decision Making. 2010;30(2):163-75. Epub 2009 Aug 12.

3. Teixeira-Pinto A, Normand, SL. Correlated bivariate continuous and binary outcomes: issues and applications. Stat Med. 2009;28(13):1753-73.

4. Fitzmaurice G, Laird, NM. Regression Models for a Bivariate Discrete and Continuous Outcome with Clustering. Journal of the American Statistical Association. 1995;90(431):845-52.

5. Thompson S, Nixon, RM. How Sensitive Are Cost-Effectiveness Analyses to Choice of Parametric Distributions? . Medical Decision Making. 2005;25(4):416-23.

6. Lunn D, Thomas A, Best N, Spiegelhalter D. WinBUGS - A Bayesian modelling framework: Concepts, structure, and extensibility. Statistics and Computing. 2000 2000/10/01;10(4):325-37. English.

7. Lunn D, Jackson, C., Best, N., Thomas, A., Spiegelhalter, D. The BUGS Book - A Practical Introduction to Bayesian Analysis: Chapman and Hall/CRC; 2012.
