# Supplementary material for: Evaluating implementation of a fire-prevention injury prevention briefing in children's centres: Cluster randomised controlled trial
Source: PLoS One. 2017 Mar 24;12(3):e0172584. doi: 10.1371/journal.pone.0172584 (PMC5365108; doi:10.1371/journal.pone.0172584)
Supplement: S2 Text — (DOCX) [file pone.0172584.s004.docx]

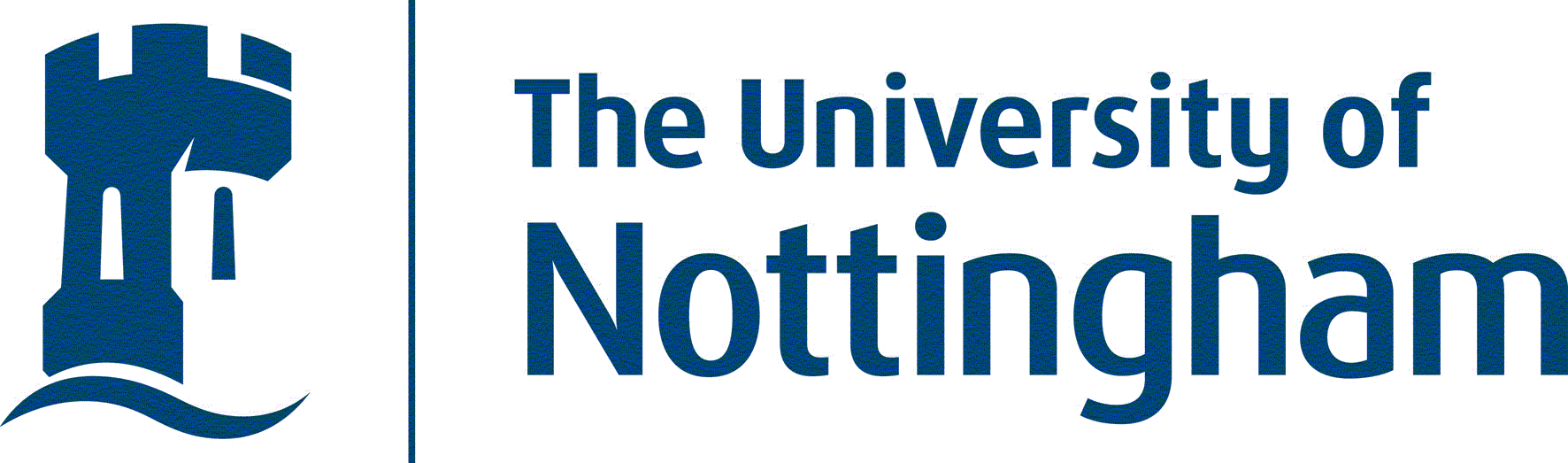


**Keeping Children Safe at Home: Randomised controlled trial of the implementation of An Injury Prevention Briefing in children’s centres for the prevention of FIRE-RELATED injuries**

**Final Version 1**

**25^th^ January 2011.**

**Short title:** KCS: RCT of implementation of fire-related injuries IPB

**Acronym:** KCS RCT

**Trial Registration:** [www.clinicaltrials.gov](http://www.clinicaltrials.gov) reference

**ISRCTN:** *complete when apply*

**NRES reference:** 11/EM0011

**Trial Sponsor:** University of Nottingham

**Funding Source:** National Institute for Health Research Programme Grants for Applied Research funding scheme (RP-PG-0407-10231)TRIAL PERSONNEL AND CONTACT DETAILS

**Sponsor:** University of Nottingham

Contact name Mr Paul Cartledge

Head of Research Grants and Contracts

Research Innovation Services

King’s Meadow Campus

Lenton Lane

Nottingham, NG7 2NR

**Chief investigator:** Professor Denise Kendrick

School of Community Health Sciences

Division of Primary Care

University of Nottingham

13^th^ floor, The Tower

University Park

Nottingham, NG7 2RD

0115 8466914

[denise.kendrick@nottingham.ac.uk](mailto:denise.kendrick@nottingham.ac.uk)

**Co-investigators:** Professor Elaine M^c^Coll

Newcastle Clinical Trials Unit

4th Floor, William Leech Building

The Medical School

Newcastle University

Framlington Place

Newcastle upon Tyne, NE2 4HH

0191 222 7260

[e.mccoll@newcastle.ac.uk](mailto:e.mccoll@newcastle.ac.uk)

Professor Elizabeth Towner,

Centre for Child & Adolescent Health

University of the West of England, Bristol

Oakfield House

Oakfield Grove

Clifton

Bristol BS8 2BN

0117 3314085

[elizabeth.towner@uwe.ac.uk](mailto:elizabeth.towner@uwe.ac.uk)

Dr Richard Reading

Clinical Research and Trials Unit Office

Level 3 East Block

Norfolk & Norwich University Hospitals NHS Foundation

Trust

Colney Lane

Norwich, NR4 7UY

01603 288476

[richard.reading@nnuh.nhs.uk](mailto:richard.reading@nnuh.nhs.uk)

Professor Alex Sutton

Medical Statistics

Room 214e, Adrian Building

University of Leicester

Leicester, LE1 7RH

0116 229 7268

[ajs22@le.ac.uk](mailto:ajs22@le.ac.uk)

**Trial Statistician:** Dr Carol Coupland

Division of Primary Care

University of Nottingham

13th Floor, The Tower

University Park

Nottingham, NG7 2RD

0115 8466916

[carol.coupland@nottingham.ac.uk](mailto:carol.coupland@nottingham.ac.uk)

**Trial Health Economist** Dr Nicola Cooper

Room 214e, Adrian Building

University of Leicester

Leicester, LE1 7RH

0116 229 7267

[njc21@leicester.ac.uk](mailto:njc21@leicester.ac.uk)

**Trial Coordinating Centre:** Professor Denise Kendrick

School of Community Health Sciences

Division of Primary Care

University of Nottingham

13^th^ floor, The Tower

University Park

Nottingham, NG7 2RD

0115 8466914

[denise.kendrick@nottingham.ac.uk](mailto:denise.kendrick@nottingham.ac.uk)

Main trial co-ordinating centre is the University of Nottingham. Other study coordinating centres are Newcastle, Bristol and Norwich.

**Trial Manager (joint):**  Ms Jane Stewart

Division of Primary Care

University of Nottingham

13th Floor, The Tower

University Park

Nottingham, NG7 2RD

01623 673323

[jane.stewart@nottingham.ac.uk](mailto:jane.stewart@nottingham.ac.uk)

**Trial Manager (joint):** Dr Toity Deave

Centre for Child & Adolescent Health

University of the West of England, Bristol

Oakfield House

Oakfield Grove

Clifton

Bristol BS8 2BN

0117 3314032

[toity.deave@uwe.ac.uk](mailto:toity.deave@uwe.ac.uk)

# SYNOPSIS

| Title | Keeping Children Safe at Home: Randomised controlled trial of the implementation of an Injury Prevention Briefing (IPB) in Children’s Centres for the prevention of fire-related injuries |
| --- | --- |
| Acronym | KCS RCT |
| Short title | KCS: RCT of implementation of fire-related injuries IPB |
| Title for study invite letters, information sheets and consent forms | Helping Children’s Centres to enhance home safety  (This title is being used on these documents to prevent raising awareness of the intervention and subsequent contamination of the control arm) |
| Chief Investigator | Professor Denise Kendrick |
| Objectives | Primary Objective  To increase the proportion of families who have a fire escape plan.  Secondary Objectives  *Family participants*   1. To test the hypothesis that providing facilitation for delivering the fire-related injury IPB to Children’s Centres is more effective, and cost-effective than providing the fire-related IPB without facilitation or than usual care in:    1. increasing the proportion of families that have smoke alarms fitted and working on every level of their home;    2. reducing the proportion of families that report fire setting or match play by their children;    3. increasing the proportion of families that have a bedtime routine for preventing fires    4. increasing the proportion of families that take part in smoking cessation courses/support;    5. increasing families’ knowledge about the causes of fires in the home;    6. increasing the proportion of families that are satisfied with home safety information provided by Children’s Centre.   *Children’s Centres as participants*   1. To assess the cost effectiveness of providing facilitation for delivering the fire-related injury IPB to Children’s Centres; 2. To assess the success of implementing the IPB in Children’s Centres; 3. To explore barriers and facilitators to implementing the IPB amongst Children’s Centre managers and staff. |
| Trial Configuration | Three arm, pragmatic, cluster randomised controlled trial with an economic analysis and a nested qualitative study. |
| Setting | Children’s Centres in the community. The unit of randomisation will be Children’s Centres. The unit of data collection will be individual Children’s Centres and also families attending those Children’s Centres. |
| Sample size estimate | 12 Children’s Centres per arm will allow an absolute difference in families with a fire escape plan of 20% to be detected with 80% power (2-sided 5% significance) for control arm prevalence of 42%, assuming an intraclass correlation coefficient of 0.05 and that outcomes are assessed on a minimum of 20 families from 33 Children’s Centre.  In order to collect outcome data on 20 families per Children’s Centre we will aim to recruit 30 families per Children’s Centre. |
| Number of participants | 36 Children’s Centres  1080 families will be recruited. |
| Eligibility criteria | First-wave Children’s Centres.  Children’s Centre staff who are responsible for the delivery of the IPB in the IPB with facilitation (I_1_) and the IPB with no facilitation (I_2_) arms.  Any family who has attended a participating Children’s Centre in the previous three months, who has a child under three years old and lives within the catchment area of that Children’s Centres. |
| Description of interventions | Intervention will include the provision of an IPB on fire-related injuries, delivered by trained Children’s Centre staff.  There are 3 arms to this RCT:  I_1_ IPB with facilitation package;  I_2_ IPB with no facilitation package;  C Usual care. |
| Duration of study | The study will take place between May 2011 and March 2014. Each family who participates in the study will be followed-up 12 months post-recruitment. Recruitment of Children’s Centres will take place May-July 2011 and families between September-November 2011. |
| Randomisation and blinding | *Randomisation*  Children’s Centres will be stratified by study coordinating centres (4 strata) and randomly allocated within strata to one of three intervention arms. Randomisation will be conducted by the Newcastle Clinical Trials Unit, using block randomisation, using permuted block randomisation, with block size of 9.  *Blinding*  It is not possible to blind Children’s Centre managers and staff or those providing the intervention to treatment arm allocation. Families will not be blinded to the intervention that their Children’s Centre provides but it is unlikely that they will be aware of intervention arms in other Children’s Centres. Analyses will be undertaken blind to treatment arm allocation. |
| Outcome measures | Family participants  *Primary outcome measure*  The primary outcome measure will be the proportion of families who have a fire escape plan.  *Secondary outcome measures*:   1. The proportion of families with smoke alarms fitted and working on every level; 2. The proportion of families that report fire setting or match play by their children; 3. The proportion of families that have a bedtime routine for preventing fires; 4. The proportion of families that take part in smoking cessation courses/support; 5. Scores of knowledge about the causes of fires in the home; 6. The proportion of families who are satisfied with home safety information provided by Children’s Centre;   The primary cost-effectiveness outcome will be the cost per family who have a fire escape plan.  Children’s Centres as participants   1. The proportion of Children’s Centres providing information and advice on the prevention of fire-related injury including fire escape planning, smoke alarms, preventing fire-setting or match play, use of bedtime routines for preventing fires, smoking cessation and referral to smoking cessation services. 2. The identification of the barriers and facilitators to Children’s Centres implementing the IPB (only in Children’s Centres in the I_1_ and I_2_ arms); 3. The success of implementing the IPB in Centres, as reported by Children’s Centres only in Children’s Centres in the I^1^ and I^2^ arms; 4. The demographic characteristics of those participating families compared with those in the catchment area of Children’s Centres. |
| Statistical methods | All analyses will be conducted on an intention-to-treat basis in that families and Children’s Centre managers and staff will be analysed in the group to which they were randomised, regardless of the intervention actually received (eg. if they moved and used a different Children’s Centre). Analyses will conform to a pre-specified analysis plan and will be undertaken blind to treatment arm allocation.  The primary outcome measure (having a fire escape plan at 12 months) will be compared between treatment arms (I_1_ vs C; I_2_ vs C and I_1_ vs I_2_) using random effects logistic regression to estimate odds ratios and 95% CI. The primary analysis will adjust for randomisation stratum (study coordinating centre) as a fixed effect and will also adjust for the lead agency of the Children's Centre (Local Authority or NHS led), having a fire escape plan at baseline and deprivation (measured using IMD2007). Sub-group analyses will explore differential effects by deprivation, measured using the IMD2007, by adding interaction terms to the regression model for the primary outcome measure. The ICC and 95% CI will be estimated for the primary outcome measure from the regression models. Models will be checked by examining level 2 residuals for normality and constant variance and level 1 and level 2 residuals for outliers.  The secondary binary outcome measures at family level will be compared between treatment arms (I_1_ vs C; I_2_ vs C and I_1_ vs I_2_) using random effects logistic regression to estimate odds ratios and 95% CI. Analyses will adjust for randomisation stratum (study coordinating centre) as a fixed effect and will also adjust for the lead agency of the Children's Centre (Local Authority or NHS led), baseline value of the outcome measure and deprivation (measured using IMD2007). The analysis of knowledge scores will be undertaken using random effects linear regression to estimate differences between treatment arms and 95% CI. If assumptions are not met, transformations will be considered and if no suitable transformation can be found, the score will be dichotomised and analysed using random effects logistic regression. Secondary outcome measures at the level of Children’s centre will be compared using logistic regression if binary and linear regression if continuous, adjusting for randomisation stratum (study coordinating centre) and lead agency of the Children's Centre (Local Authority or NHS led).  A complete case analysis will be undertaken. If any Children’s Centres are lost to follow-up we will consider a range of approaches to undertaking sensitivity analyses to assess the robustness of the findings.  No interim analyses will be undertaken.  To assess the cost-effectiveness of implementing an IPB in Children’s Centres for preventing fire-related injuries: firstly, the resource-use data collected throughout the trial will be combined with unit cost information and summed together to obtain an average cost per person (together with its uncertainty) for each intervention. Secondly, these cost data will be combined with the clinical effectiveness data (e.g. proportion of families who have a fire escape plan) to inform an incremental cost-effectiveness analysis. Sensitivity analysis will be undertaken to assess the robustness of the results to any assumptions made in the analysis. |
| Qualitative study | Twelve months after the start of the intervention, information about the barriers and facilitators to implementing the intervention will be collected. This is an important aspect to the trial to be able to explore the process of implementing the intervention, how the study and the intervention fit into the context of Children’s Centres, the unmet needs of the Children’s Centre managers and staff, levels of adherence to the intervention and for whom the intervention appeared to work best. This will be in the form of semi-structured interviews of a purposive sample of Children’s Centre managers in the IPB plus facilitation (I_1_) and the IPB without facilitation (I_2_) arms. A maximum of 18 interviews will be undertaken. We will use a purposive sample of Centres in the two intervention arms in each study area to obtain Children’s Centres with a range of characteristics including extent of implementation of the IPB. |

#

# ABBREVIATIONS

| AE | Adverse Event | |  |
| --- | --- | --- | --- |
| CI  CRF | Chief Investigator  Case Report Form | |  |
| CV  DAP | | Curriculum Vitae  Data Analysis Plan | |
| ED  EOT  GCP  ICF  IPB | | Emergency Department  End of Trial  Good Clinical Practice  Informed Consent Form  Injury Prevention Briefing | |
| NHS | | National Health Service | |
| NIHR  NRES  PARIHS | | National Institute for Health Research  National Research Ethics Service  Promoting Action on Research in Health Services (framework) | |
| PCT | | Primary Care Trust | |
| PI  PIS  REC | | Principal Investigator  Participant Information Sheet  Research Ethics Committee | |
| R&D  TSC | | Research and Development  Trial Steering Committee | |
| UIC  UWE | | Unique Identity Code  University of the West of England, Bristol | |
| AE | | Adverse Event | |

**TABLE OF CONTENTS**

[TRIAL / STUDY PERSONNEL AND CONTACT DETAILS 2](#_Toc234212244)

[SYNOPSIS 5](#_Toc234212245)

[ABBREVIATIONS 9](#_Toc234212246)

[TRIAL / STUDY BACKGROUND INFORMATION AND RATIONALE 12](#_Toc234212247)

[TRIAL / STUDY OBJECTIVES AND PURPOSE 13](#_Toc234212248)

[PURPOSE 13](#_Toc234212249)

[PRIMARY OBJECTIVE 13](#_Toc234212250)

[SECONDARY OBJECTIVES 14](#_Toc234212251)

[TRIAL / STUDY DESIGN 14](#_Toc234212252)

[TRIAL / STUDY CONFIGURATION 14](#_Toc234212253)

[Primary endpoint 14](#_Toc234212254)

[Secondary endpoint 14](#_Toc234212255)

[Safety endpoints 15](#_Toc234212256)

[Stopping rules and discontinuation 16](#_Toc234212257)

[RANDOMIZATION AND BLINDING 16](#_Toc234212258)

[Maintenance of randomisation codes and procedures for breaking code 16](#_Toc234212259)

[TRIAL MANAGEMENT 16](#_Toc234212260)

[DURATION OF THE TRIAL / STUDY AND PARTICIPANT INVOLVEMENT 17](#_Toc234212261)

[End of the Trial 17](#_Toc234212262)

[SELECTION AND WITHDRAWAL OF PARTICIPANTS 17](#_Toc234212263)

[Recruitment 17](#_Toc234212264)

[Inclusion criteria 18](#_Toc234212265)

[Exclusion criteria 18](#_Toc234212266)

[Expected duration of participant participation 18](#_Toc234212267)

[Removal of participants from the trial 18](#_Toc234212268)

[Informed consent 19](#_Toc234212269)

Data Collection 19

[TRIAL / STUDY TREATMENT AND REGIMEN 21](#_Toc234212270)

Facilitation of the IPB and c[ompliance with the intervention](#_Toc234212271) 21

[Criteria for terminating trial 22](#_Toc234212272)

[STATISTICS 22](#_Toc234212273)

[Methods 22](#_Toc234212274)

[Sample size and justification 23](#_Toc234212275)

[Assessment of efficacy 24](#_Toc234212276)

[Assessment of safety 25](#_Toc234212277)

[Procedures for missing, unused and spurious data 25](#_Toc234212278)

[Definition of populations analysed 25](#_Toc234212279)

[ADVERSE EVENTS 25](#_Toc234212280)

[ETHICAL AND REGULATORY ASPECTS 26](#_Toc234212286)

[ETHICS COMMITTEE AND REGULATORY APPROVALS 26](#_Toc234212287)

[INFORMED CONSENT AND PARTICIPANT INFORMATION 26](#_Toc234212288)

[RECORDS 26](#_Toc234212289)

[Case Report Forms 26](#_Toc234212290)

[Direct access to source data / documents 28](#_Toc234212292)

[DATA PROTECTION 28](#_Toc234212293)

[QUALITY ASSURANCE & AUDIT 28](#_Toc234212294)

[INSURANCE AND INDEMNITY 28](#_Toc234212295)

[TRIAL CONDUCT 29](#_Toc234212296)

[TRIAL DATA 29](#_Toc234212297)

[RECORD RETENTION AND ARCHIVING 29](#_Toc234212298)

[DISCONTINUATION OF THE TRIAL BY THE SPONSOR 29](#_Toc234212299)

[STATEMENT OF CONFIDENTIALITY 29](#_Toc234212300)

[PUBLICATION AND DISSEMINATION POLICY 30](#_Toc234212301)

[USER AND PUBLIC INVOLVEMENT 30](#_Toc234212302)

[STUDY FINANCES 30](#_Toc234212303)

[Funding source 30](#_Toc234212304)

[Participant stipends and payments 30](#_Toc234212305)

[SIGNATURE PAGES 31](#_Toc234212306)

[REFERENCES 33](#_Toc234212307)

STUDY FLOW CHART 34

#

# BACKGROUND INFORMATION AND RATIONALE FOR CONDUCTING THE TRIAL

Unintentional injury is one of the leading causes of death in children aged 1-4 years^1^ and is therefore a major public health challenge facing pre-school children in England today. Child fire deaths are commonly caused by smokers’ materials (e.g. cigarettes, cigars and tobacco), and cigarette lighters and matches, respectively. Fire-related deaths and injuries disproportionately affect the disadvantaged^2^. In the UK, children whose parents had never worked or were long-term unemployed have death rates from exposure to smoke, fire and flames 37 times higher than those of children whose parents had managerial/professional occupations^3^. The UK does not compare well with other high-income countries for fire-related injuries, having one of the highest fatality rates for deaths from fire and flames in children aged 0–14 years^4^. It is estimated that 90% of severe injuries in this age group are potentially preventable^5^.

All children are vulnerable to injury because their physical, psychological and behavioural characteristics place them at risk in a largely adult world. The patterns and types of injury are closely linked with child development. Furthermore, the social construction of the adult world makes the children of the poor even more vulnerable to injury. The number of unintentional injury deaths has been declining recently, but steep socio-economic gradients persist at the start of the 21st century. According to the 2001 census, around 5% of children in England and Wales were classified as having parents who had never worked or were long-term unemployed, but they accounted for 33% of injury deaths in 2001-3^3^.

Over the years, the importance of child injury prevention has been emphasised in Government policies related to health, inequalities and children’s issues. ‘Saving Lives: Our Healthier Nation’, set national targets to reduce deaths and serious injury by 2010.^6^ Unintentional injury has been recognised as an inequalities issue and actions of government departments to tackle unintentional injuries are emphasised in the Public Health White Paper ‘Choosing Health: making healthier choices easier’ and delivery plan^7^ The Accidental Injury Task Force, coordinated by the Department of Health, reported in 2002, recommending action at all levels within the NHS^8^. The ‘National Service Framework for children’ included a specific set of recommendations relating to preventing unintentional injuries and reducing injury inequalities^9^ The ‘Staying Safe Action Plan’ highlighted the role of health visiting services in injury prevention and introduced a new national home safety equipment scheme targeted at disadvantaged families^10^. ‘Every Child Matters’ emphasised the importance of safety in one of it's five overall aims^11^ and ‘The Children Act’ set out the responsibilities of Safeguarding Children Boards, one priority of which relates to the prevention of unintentional injuries and deaths^12^. Whilst these policies are being reviewed by the Coalition government, the recent White Paper “Healthy Lives, Healthy People” indicates commitment to improving child public health with emphasis on early intervention and prevention, with an increased focus on disadvantaged families and increased investment in health visiting^13^.

Those who are charged with developing and implementing strategies to prevent unintentional injury at a local level often find it difficult to do so. The 2007 Audit Commission/Healthcare Commission report ‘Better Safe than Sorry’, emphasised there is no single, clear cross-governmental statement, drawing together what needs to be done to reduce unintentional injury^14^. At a local level in the NHS, the report found little evidence of a systematic approach to develop, implement and monitor programmes to prevent unintentional injuries in children. This highlights the importance of developing and implementing evidence based child injury prevention within the NHS.

We are in receipt of programme grant funding from the National Institute for Health Research to undertake a series of inter-linked studies addressing the effectiveness and cost effectiveness of a range of interventions to prevent falls, poisoning and thermal injuries (scalds and fire-related injuries) in pre-school children, to translate the findings of this research into practice and to evaluate that translation process. This will involve a randomised controlled trial which will take place in Children’s Centres. These are hubs where children under five years old and their families can receive seamless integrated services and information. Recent policy documents indicate that Children’s centres will focus particularly on engaging with families where children are at risk of poor outcomes^13^ and these are likely to be families who are also at greatest risk of fire-related injuries.

The Injury Prevention Briefing (IPB), which forms the basis of the intervention, brings together the scientific evidence on what works, or can be regarded as best practice, with the practical experience of people who already run injury prevention programmes in the field, both through Children’s Centres or elsewhere. Different sources of evidence generated from other work streams within this NIHR-funded programme of research have been used to prepare the IPB. These include:

- Systematic reviews of what interventions work in preventing injuries from house fires and what health promotion approaches work with families of preschool children, and decision-analyses related to the prevention of house fires.
- Postal surveys and face-to-face interviews with Children’s Centre managers about injury prevention initiatives in Children’s Centres.
- Interviews with parents of pre-school children about their fire-related practices in the home e.g. their ownership and maintenance of smoke alarms, whether they have prepared a fire escape plan.
- Interviews with ‘key informants’ about national policy in this field.
- Workshops with local practitioners and policy makers about how to implement programmes in Children’s Centres and how to reach families in the community.

The specific research question for this part of the programme is:

How effective and cost-effective is the implementation of an Injury Prevention Briefing (IPB) in Children’s Centres for preventing fire-related injuries?

# TRIAL OBJECTIVES AND PURPOSE

## PURPOSE

To conduct a randomised controlled trial to assess the effectiveness and cost-effectiveness of implementing an Injury Prevention Briefing in Children’s Centres for preventing fire-related injuries.

## PRIMARY OBJECTIVE

To test the hypothesis that providing facilitation for delivering the fire-related injury IPB to Children’s Centres is more effective, and cost-effective, than providing the fire-related IPB without facilitation or than usual care in increasing the proportion of families attending Children’s Centre who have a fire escape plan. The primary objective relates to fire escape planning as there was evidence from the systematic reviews and decision analyses described above that interventions can be effective and cost effective in increasing fire escape planning by families. Although the best evidence exists for interventions to promote smoke alarm use, this was not chosen as the primary outcome measure due to the high prevalence of functioning smoke alarms (> 90%) found in the interviews with parents attending Children’s Centres in an earlier piece of work within the programme.

## SECONDARY OBJECTIVES

Family participants

1. To test the hypothesis that providing facilitation for delivering the fire-related injury IPB to Children’s Centres is more effective, and cost-effective than providing the fire-related IPB without facilitation or than usual care in:
   1. increasing the proportion of families who have smoke alarms fitted and working on every level of their home;
   2. reducing the proportion of families who report fire setting or match play by their children;
   3. increasing the proportion of families who have a bedtime routine for preventing fires
   4. increasing the proportion of families who take part in smoking cessation courses/support;
   5. increasing families’ knowledge about the primary causes of fires in the home;
   6. increasing the proportion of families who are satisfied with home safety information provided by Children’s Centre.

Children’s Centres as participants

1. To assess the cost effectiveness of providing facilitation for delivering the fire-related injury IPB to Children’s Centres;
2. To assess the success of implementing the IPB in Children’s Centres;
3. To explore barriers and facilitators to implementing the IPB amongst Children’s Centre managers and staff.

##

## TRIAL DESIGN

## TRIAL CONFIGURATION

This is a multi-centre (Nottingham, Newcastle, Norwich and Bristol) cluster randomised trial with an economic analysis and a nested qualitative study. All analyses will be conducted on an intention-to-treat basis in that families and Children’s Centre managers and staff will be analysed in the group to which they were randomised, regardless of the intervention actually received.

### Primary endpoint

Family participants

The primary outcome measure will be the proportion of families who have a fire escape plan (ascertained from self-completion questionnaire).

### Secondary endpoint

Secondary outcome measures (also ascertained from self-completion questionnaire) will include:

1. The proportion of families with smoke alarms fitted and working on every level;
2. The proportion of families who report fire setting or match play by their children;
3. The proportion of families who have a bedtime routine for preventing fires;
4. The proportion of families who take part in smoking cessation courses/support;
5. Score for families’ knowledge of the causes of fires in the home;
6. The proportion of families who are satisfied with home safety information provided by Children’s Centre;
7. Families’ resource-use and expenditure in the I_1_ and I_2_ arms incurred as a result of the intervention.

Children’s Centres as participants

1. The proportion of Children’s Centres providing information and advice on the prevention of fire-related injury including: fire escape planning, smoke alarms, preventing fire-setting or match play, use of bedtime routines for preventing fires, smoking cessation and referral to smoking cessation services (ascertained from Children’s Centre self-completion questionnaire);
2. The identification of the barriers and facilitators to Children’s Centres implementing the IPB (only in Children’s Centres in the I_1_ and I_2_ arms) (ascertained from interviews with Children’s Centre managers/staff);
3. A comparison of the resource-use and expenditure incurred as a result of the intervention in the I_1_ and I_2_ arms (ascertained from Children’s Centre self-completion questionnaire);
4. The success of implementing the IPB in Children’s Centres (only in Children’s Centres in the I_1_ and I_2_ arms) (ascertained from self completion implementation measurement tool based on the PARIHS framework^16^ and telephone and face-face contacts with research team at months 1, 3, 4-5 and 8);
5. The demographic characteristics of those participating families compared with those in the catchment area of Children’s Centres (ascertained from baseline questionnaire from participating families and data routinely collected by Children’s Centres).

**Health economic endpoints**

The primary cost-effectiveness outcome will be the cost per family who have a fire escape plan.

Secondary economic outcomes will include:

- Cost per family with smoke alarms fitted and working on every level;
- Cost per family that report fire setting or match play by their children;
- Cost per family that have a bedtime routine for preventing fires;
- Cost per family that take part in smoking cessation courses/support;
- Cost per family that demonstrate knowledge about the primary causes of fires in the home;
- Cost per family that is satisfied with home safety information provided by Children’s Centre.

###

### Safety endpoints

N/A

### Stopping rules and discontinuation

## We do not anticipate any need for premature stopping of the trial for safety concerns. Interim analysis is not planned, and therefore we do not anticipate early discontinuation for futility.

##

## RANDOMISATION AND BLINDING

*Randomisation*

Children’s Centres will be stratified by study coordinating centre (4 strata) and randomly allocated within strata to one of three intervention arms. Randomisation will be conducted by the Newcastle Clinical Trials Unit, using permutable block randomisation, with block size of 9. The allocation code will be will be stored at Newcastle Clinical Trials Unit and only they and each study coordinating centre research team will have access to it. To ensure concealment of allocation each study coordinating centre will only be notified of the allocation of a given Children’s Centre in their area to trial arm once that Centre, and the families within it, have been recruited to the study as described below. This intervention is in addition to information that is already being offered within Children’s Centres. This will minimise post-randomisation recruitment bias. Therefore, parents in both control (‘usual’ care) and intervention Children’s Centres may have received fire-related safety information prior to being allocated to one of the three arms. Parents not recruited to the study will receive whatever information and support that Children’s Centre routinely provides. This may include additional information provided as part of the study. This will be dependent on how Children’s Centres organise the delivery of the IPB. For example, if they offer sessions on preventing house fires they will invite any parent who would normally be invited to these sessions since they will be seen as sessions that a Children’s Centre would provide as part of their ‘usual care’.

The research team in each study coordinating centre will ask Children’s Centres to participate. Once a Children’s Centre has agreed to take part, families from that Children’s Centre will start to be recruited. When the required number of families has been recruited that Centre will be allocated to a treatment arm. To avoid post randomisation recruitment bias, randomisation will only take place after a Children’s Centre and the families within that Children’s Centre have been recruited.

*Blinding*

It is not possible to blind Children’s Centre managers and staff or those providing the intervention to treatment arm allocation. Families will not be blinded to the intervention that their Children’s Centre provides but it is unlikely that they will be aware of the intervention being provided in other Children’s Centres. Analyses will be undertaken blind to treatment arm allocation.

### Maintenance of randomisation codes and procedures for breaking code

N/A

## TRIAL MANAGEMENT

The Chief Investigator (Denise Kendrick) has overall responsibility for the trial and shall oversee all trial management. There will be two trial managers (Jane Stewart, Nottingham and Toity Deave, Bristol) who will undertake, jointly, the day-to-day management of the trial. The R&D manager of Nottinghamshire County Teaching PCT (Rachel Illingworth) will have responsibility for financial monitoring and reporting. A study management group (for the NIHR programme grant) comprising all NIHR programme grant co-applicants plus research staff employed to work on all the studies meets three monthly during the duration of the five year programme grant, which will cover the duration of this trial. The trial managers (Jane Stewart and Toity Deave) will arrange and manage minuted, telephone conferences every six weeks with researchers in the four centres to ensure consistency of trial process and address queries. Each centre will establish monthly minuted meetings between the PI and the researchers employed on the study.

A Trial Steering Committee (TSC) will be established to oversee the trial. It will meet twice a year with email communication in between the meetings if the independent TSC Chair deems it necessary. The TSC will decide on the appropriateness of establishing a separate data monitoring and ethics committee (DMEC). The TSC will report to the study management group, and to the NIHR via its required interim reports.

## DURATION OF THE TRIAL AND PARTICIPANT INVOLVEMENT

The trial will take place between May 2011 and March 2014. Recruitment of Children’s Centres will take place between May-July 2011 and of families between September-November 2011. Each family that participates in the study will be followed-up 12 months post-recruitment.

### End of the Trial

The trial will be completed when the 12 month follow-up data collection has taken place and the data collection for the implementation process at each Children’s Centre has been completed. Families’ involvement with the trial will finish once the follow-up data collection for the last family has been completed. Children’s Centre managers’ and staff involvement will finish once the implementation process data have been completed and the last interview has been undertaken with Children’s Centre managers and staff.

## SELECTION AND WITHDRAWAL OF PARTICIPANTS

### Recruitment

Children’s Centres

Children’s Centres in the four study coordinating centres who are based in the two or three Primary Care Trusts (PCTs) closest to the study coordinating centres (36 Children’s Centres in 11 PCTs), and who were First-wave Children’s Centres, will be invited to take part in the study by postal invitation followed by a telephone call. The postal invitation will include the study information leaflet, originating from the study coordinating centre research team and the Child Accident Prevention Trust. This will be followed-up by telephone contact with Children’s Centre manager, or a visit to the Children’s Centre, to discuss the study. Those interested in taking part will be sent (or in the case of a visit to a Children’s Centre, will be given) a consent form to complete and return. If more than nine Children’s Centre in each study coordinating centre area agree to take part, nine will be randomly selected.

Families

Families who fit the inclusion criteria will be identified from databases held by Children’s Centres and recruited from the 36 participating Children’s Centres in the four study coordinating centres. There will be several strategies for making the initial approach, depending on which the Children’s Centre staff considers will work best for their population. Combinations of strategies may be used in some centres. Information about the study will be displayed in the relevant areas in each Children’s Centre and each Children’s Centre will use its usual forms of communication to inform families attending the Centre about the trial. This may take the form of posters within the entrance area or specific areas within the Children’s Centre or verbal communication.

1. A study invitation letter, participant information sheet (PIS), baseline questionnaire and a consent form will be posted to parents by Children’s Centre staff. Families will be able to discuss the study with a member of the research team by telephone, or in person, if a researcher is present in the Children’s’ centre at the families next attendance. Families for whom the Children’s Centre would normally use an interpreter will not be sent study invitation letters, instead Children’s Centre staff will be asked to invite these families to the study during an attendance at the Centre when an interpreter is available;
2. A member of the Children’s Centre staff will give parents attending the Centre a study invitation letter, participant information sheet (PIS), baseline questionnaire and a consent form. Parents will have the study explained to them and will be given the opportunity to discuss the study and ask any questions they would like to prior to completing and returning the consent form. This may be in person with the researcher at an arranged time or by telephone;
3. A member of the Children’s Centre staff will approach parents in the Children’s Centre. Parents will have the study explained to them and be given a study invitation letter, participant information sheet (PIS), baseline questionnaire and a consent form. Parents will be given the opportunity to discuss the study with the researcher who will be present within the Children’s Centre and ask any questions they would like to prior to completing and returning the consent form. Where participants are happy to complete the consent form at this point, parents will be invited to complete the questionnaire whilst the researcher and Children’s Centre staff are present. If they do not wish to do this they will be provided with a freepost envelope to return the consent form and completed questionnaire.

If needed, the usual Children’s Centre interpreter and translator services will be available to assist with discussion of the trial, the participant information sheets and consent forms. The consent forms and information sheets will not be available printed in other languages. Participants will only be considered to be recruited to the trial if they complete a consent form and the baseline questionnaire.

### Inclusion criteria

Children’s Centres:

- First-wave Children’s Centres in the four study areas (Nottingham, Newcastle, Bristol and Norwich).

Children’s Centre staff

- Children’s Centre staff who are responsible for the delivery of the IPB in the IPB plus facilitation (I_1_) and the IPB without facilitation (I_2_) arms.

Families

- Any family who has attended the participating Children’s Centre in the previous three months, who have a child under three years old, and lives within the catchment area of that Children’s Centres.

### Exclusion criteria

Children’s Centres:

- Children’s Centres that are not a First-wave Children’s Centre.

Families

- Families who attend a participating Children’s Centre who do not have any children under the age of 3 years
- Any parent who is under-16 years old.

### Expected duration of participant participation

### Children’s Centres will be participating in the trial for 28 months and families will participate for 18 months.

### Removal of participants from the trial

Children’s Centre and family participants may be withdrawn from the trial at their own request. They will be made aware that this will not affect their future care or support from their Children’s Centre. Participants, both Children’s Centres and families, will be made aware (via the information sheet and consent form) that should they withdraw the data collected to date cannot be erased and may still be used in the final analysis. Children’s Centres that withdraw prior to commencement of the intervention will be replaced. Other premature withdrawals will not be replaced.

### Informed consent

The process for obtaining participant informed will be in accordance with the REC guidance, Good Clinical Practice (GCP) and any other regulatory requirements that might be introduced. Parents recruited face-to-face in Children’s Centres will have the trial explained to them by a member of the research team or Children’s Centre staff and will be given a PIS. Parents will be able to ask the researcher any questions concerning participation in the study or be given the research team contact details. Participants will be able to sign the informed consent form (ICF) at this time, or take it home and sign at a later date and return to the study team by post. Families recruited postally will be given the telephone number of the research team whom they can contact to discuss the study and have an opportunity to ask questions about the study. They can complete, sign and date the ICF at a time of their choosing and return it to the researchers in a freepost envelope. All ICFs will be signed and dated by the parent and the researcher. The original ICF will be kept by the study coordinating centre in the trial master file and a copy will be sent to/kept by each participant. ICFs returned by post will be signed and dated by the researcher prior to the copy being sent to the parent. ICFs will be completed prior to the delivery of any intervention. On receipt of an ICF the relevant Children’s Centre will be informed of that family’s participation.

Children’s Centre managers will be sent a PIS about the trial, with information that relates to both the Centre’s anticipated involvement and the family participants. They will have the opportunity to discuss the study by telephone or face-face with a member of the research team and be given the opportunity to ask any questions about the trial. They will be asked to sign an ICF, which will then be signed by the researcher. The original ICF will be kept by the study coordinating centre in the trial master file and a copy will be sent to/kept by each participating Children’s Centre. ICFs returned by post will be signed by the researcher prior to the copy being sent to the Children’s Centre. ICFs will be completed prior to randomisation. Each Children’s Centre manager and/or staff who are interviewed to explore barriers and facilitators to implementing the IPB will complete, sign and date a separate ICF prior to the interview. Two copies will be completed and signed and dated by the researcher, one copy will be kept by the interviewee and the other will be kept by the study coordinating centre in the trial master file.

Should there be any subsequent amendment to the protocol, which might affect a participant’s participation in the trial, the investigator shall follow all applicable regulatory requirements pertaining to approval of the amended Consent Form by the REC and use of the amended form (including for on-going participants). Continuing consent will be obtained using an amended Consent Form which will be signed by the participant.

Data collection

**Family participants**

Baseline and follow-up data will be collected by self-completion questionnaire. Baseline questionnaires will be given to family participants when they attend Children’s Centres by the researchers from each of the four study coordinating centres or by the relevant Children’s Centre staff, or will be posted by Children’s Centre staff. Questionnaires can be self-completed whilst at the Children’s Centre and handed to researchers or Children’s Centre staff, taken home and completed, or completed wherever the parents prefer and returned to researchers using freepost envelopes. One reminder will be sent to any parent who does not return the questionnaire. Follow-up data and the reminder, if necessary, will be collected by postal, telephone, home visit, or at Children’s Centres, which ever method is preferred by the participant. If necessary, a home visit will be undertaken to help completion of a questionnaire. Mode of administration will be recorded. Data will be entered onto identical databases at each study coordinating centre and stored on NHS or University servers. Once data collection is completed, data will be merged onto one database prior to analysis. At the end of the trial, data from all centres will be archived by the Nottingham centre for a period of seven years as set out in the University of Nottingham’s Code of Research Conduct.

The questionnaires are in the process of being developed and will be sent to the REC prior to being used within the study. Questionnaires will include questions on socio-demographic information about the household, previous fire-related accidents, fire safety behaviours and fire-safety equipment, knowledge and understanding of what causes fires, home safety information provided by Children’s Centres and their perception of the effectiveness of this information. The follow –up questionnaire will be identical to the baseline questionnaire except it will not collect socio-demographic data and it will collect data about resource-use and expenditure incurred as a result of the intervention; i.e. travel costs to attend educational sessions, equipment purchased and services attended. (e.g. smoking cessation). Parents returning completed questionnaires will be sent £5 gift vouchers for use in local stores as previous systematic reviews show small monetary incentives increase response rates^15^. Questionnaires will be developed and piloted with families between January – April 2011.

**Children’s Centres as participants**

Data will be collected on the proportion of Children’s Centres providing information and advice on the prevention of fire-related injury. This will include fire escape planning, smoke alarms, preventing fire-setting or match play, use of bedtime routines for preventing fires, smoking cessation and referral to smoking cessation services. These data will be collected from Children’s Centre managers, in all trial arms, via postal, telephone or email questionnaire at baseline and at 12 months post commencement of the intervention in the IPB plus facilitation (I_1_) and the IPB without facilitation (I_2_) arms and at 12 months post randomisation in the control arm. Mode of administration will be recorded. Questionnaires will be developed and piloted with Children’s Centres managers between January – April 2011.

Children’s Centre managers and staff in the IPB plus facilitation (I_1_) and the IPB without facilitation (I_2_) arms will have telephone and face-face contact with the research team to explore the success of implementing the IPB at months 1, 3, 4-5 and 8 (see page 22 for details). Implementation successes and difficulties will be recorded at each contact.

Children’s Centre managers and staff in the IPB plus facilitation (I_1_) and the IPB without facilitation (I_2_) arms will also be asked to complete a paper-based implementation measurement tool (which will be developed between Jan – April 2001 and will be based on The PARIHS (Promoting Action on Research in Health Services)^16^ framework to explore the success of the implementation process. This will take place at the end of the 12 month intervention period. The PARIHS framework is an evaluation instrument to explore the effectiveness of implementation strategies. This includes three core elements that determine the success of research implementation in each of which there are several components:

1. Evidence – the strength and nature of the evidence as perceived by multiple stakeholders. The four components relate to the different sources of evidence.
2. Context – the quality of the context or environment in which the research is implemented. The three components relate to the organisational culture, leadership and the organisations evaluation.
3. Facilitation – processes by which implementation is facilitated. The three components relate to facilitation, firstly as enabling, secondly as responsive and interactive, and thirdly as internal or external.

At the end of the 12 month intervention period, information about the barriers and facilitators to implementing the intervention will be collected using a nested qualitative study. This is an important aspect to the trial to be able to explore the process of implementing the intervention, how the study and the intervention fit into the context of Children’s Centres, the unmet needs of the Children’s Centre managers and staff, levels of adherence to the intervention and for whom the intervention appeared to work best, This will be in the form of semi-structured interviews (telephone or face-to-face) of Children’s Centre managers and/or staff responsible for the delivery of the IPB in the IPB plus facilitation (I_1_) and the IPB without facilitation (I_2_) arms. We will use a purposive sample of Centres in the two intervention arms in each study area (maximum of 18 interviews). This will allow us to get a range based on various characteristics including implementation of the IPB, which will be assessed based on the telephone and face-face contacts with the research team (described on page 22) and the implementation measurement tool devised using the PARIHS framework^16^.

The interview guide will be developed using the results of the systematic review of the barriers and facilitators for home injury prevention interventions, the interviews that were undertaken with Children’s Centre managers and staff in earlier parts of the programme of research and the PARIHS framework. This will cover the following areas:

- Children’s Centre manager’s/staff’s overall perception of the intervention
- Who delivered the intervention eg. Children’s Centre staff, health visitor
- What were the barriers to implementing the intervention (covering the 3 core elements in the PARIHS framework)
- What were the facilitators to implementing the intervention (covering the 3 core elements in the PARIHS framework)
- The resources used to implement the intervention
- Other issues that the Children’s Centre manager/staff want to raise

Recording, transcribing and security of data

Consent forms and questionnaires will be treated as confidential documents and held securely at each study coordinating centre and all identifiable information will be removed once they are received. The interviews will be digitally audio-recorded with the consent of the participant and the participant will be identified only by their UIC. They will be anonymised and stored with the research data as source data. Audio-recordings will be transcribed verbatim. Data will be entered onto identical databases at each study coordinating centre and stored on password protected NHS or University servers. At the end of the trial, data from all centres will be archived by the Nottingham centre for a period of seven years as set out in the University of Nottingham’s Code of Research Conduct.

## TRIAL TREATMENT AND REGIMEN

The intervention will include the provision of an IPB on fire-related injuries, and facilitation to implement the briefing. Any Centre allocated to the control arm, ‘usual care’, will continue to use health promotion guidelines if they are already using them. A schematic diagram of the trial and the IPB is attached to this protocol.

There are 3 arms to this RCT:

I_1_ = Provision of IPB and facilitation for delivering it;

I_2_ = Provision of IPB without facilitation;

C =Usual care.

### Facilitation of the IPB and compliance with the intervention

A facilitation package will be designed to ensure as consistent an approach to facilitation within each intervention Children’s Centre as possible. Discussions will take place between each Fire & Rescue Service local to the four study coordinating centres about the IPB and its implementation. The package will comprise the following:

1. Training session run jointly between the local research team and the Fire & Rescue Service. This will be followed by a meeting run by the same team to design a plan for implementing the IPB in each Children’s Centre and Centres will be expected to provide at least one fire safety session for all parents taking part in the study.
2. One month follow-up telephone contact from the research team to monitor progress and answer any questions;
3. Three month face-to-face meeting between Children’s Centre and research team to monitor progress;
4. Additional meeting at 4-5 months if any Children’s Centre in a study coordinating centre is experiencing difficulties or problems in implementing the IPB;
5. Eight month follow-up telephone contact to monitor progress.

Each contact with a Children’s Centre will be recorded by each research team, together with relevant field notes, on a form and retained within the study notes in each study coordinating centre. Compliance with the intervention will be assessed from the telephone and face to face contacts.

Potential risks and burdens for research participants.

We do not anticipate any potential risks to study participants. The main burden for Children’s Centres will be the time taken to complete questionnaires, undertake the interview and the self-completion tools (see above for details). For family participants, the main obligation will be the time taken to complete the questionnaires (baseline and follow-up). For those families in the IPB with facilitation arm there will also be the time taken to attend between 1 - 5 fire safety sessions at the Children's Centre.

If any participants have suffered a house fire or has had relatives of friends injured in one this may cause distress when parents complete questionnaires. Researchers will be trained to respond sensitively in such situations for example when discussing the study with parents or helping them to complete questionnaires. Specific advice is given in the IPB for Children's Centres about undertaking fire safety sessions in such circumstances.

It is possible that during any home visits that researchers may be told or become aware of child protection issues. All researchers undertaking home visits will be trained in child protection and will follow local safeguarding procedures.

###

### Criteria for terminating trial

There are no adverse events anticipated and no interim analyses will be undertaken. The TSC will decide on the appropriateness of establishing a separate data monitoring and ethics committee (DMEC) should an adverse event occur.

**ANALYSES**

# Statistics

### Methods

An analysis plan will be produced by the trial statistician and health economist, who will oversee and/or undertake the statistical and economic analyses.

All analyses will be conducted on an intention-to-treat basis in that families and Children’s Centre managers and staff will be analysed in the group to which they were randomised, regardless of the intervention actually received (eg. if the family moved and used a different Children’s Centre). Analyses will conform to a pre-specified analysis plan and will be undertaken blind to treatment arm allocation.

Characteristics of Children’s Centres and families at baseline will be compared informally between the three treatment arms. The primary outcome measure (having a fire escape plan at 12 months) will be described using frequencies and percentages and compared between treatment arms (I_1_ vs C; I_2_ vs C and I_1_ vs I_2_) using random effects logistic regression to estimate odds ratios and 95% CI. This analysis will have families at level one and Children’s Centres at level two, to allow for clustering of responses within Children's Centres. The primary analysis will adjust for randomisation stratum (study coordinating centre) as a fixed effect and will also adjust for the lead agency of the Children's Centre (Local Authority or NHS led), having a fire escape plan at baseline and deprivation (measured using IMD2007 obtained from families’ postcode). Deprivation has been specified as it is strongly associated with house fire injuries^2,3^ and measures of deprivation have previously been found to be associated with the effectiveness of home injury prevention interventions^18^.

The ICC and 95% CI will be estimated for the primary outcome measure from the regression models. Models will be checked by examining level 2 residuals for normality and constant variance and level 1 and level 2 residuals for outliers.

Sub-group analyses will explore differential effects by deprivation, measured using the IMD2007, by adding interaction terms to the regression model for the primary outcome measure. IMD will be entered as a continuous covariate if there is a linear relationship with outcome.

The secondary binary outcome measures at family level will be described using frequencies and percentages and compared between treatment arms (I_1_ vs C; I_2_ vs C and I_1_ vs I_2_) using random effects logistic regression to estimate odds ratios and 95% CI, allowing for clustering of responses within Children's Centres. The analyses will adjust for randomisation stratum (study coordinating centre) as a fixed effect and will also adjust for the lead agency of the Children's Centre (Local Authority or NHS led), baseline value of the outcome measure and deprivation (measured using IMD2007 obtained from families’ postcode). The analysis of knowledge scores will be undertaken using random effects linear regression to estimate differences between treatment arms and 95% CI, allowing for clustering of responses within Children's Centres. A residuals analysis will be carried out to check the assumptions of the analysis, and if they are invalid transformations will be considered and if no suitable transformation can be found the score will be dichotomised and analysed using random effects logistic regression. Secondary outcome measures at the level of Children’s centre will be compared using logistic regression if binary and linear regression if continuous, adjusting for randomisation stratum (study coordinating centre) and lead agency of the Children's Centre (Local Authority or NHS led).

A complete case analysis will be undertaken. If any Children’s Centres are lost to follow-up we will consider a range of approaches to undertaking sensitivity analyses to assess the robustness of the findings.

No interim analyses will be undertaken.

Health economic analysis

To assess the cost-effectiveness of implementing an Injury Prevention Briefing in Children’s Centres for preventing fire-related injuries, firstly, resource-use data collected throughout the trial will be combined with unit cost information and summed to obtain an average cost per person (together with its uncertainty) for each intervention. Secondly, these cost data will be combined with the clinical effectiveness data (e.g. proportion of families who have a fire escape plan) to inform the incremental cost-effectiveness analysis. Sensitivity analysis will be undertaken to assess the robustness of the results to any assumptions made in the analysis.

### Sample size and justification

11 Children’s Centres per arm will allow an absolute difference in the percentage of families with a fire escape plan of 20% in arm I_1_ or arm I_2_ compared to the control arm to be detected. This assumes a control arm prevalence of 42% (obtained from interviews with parents in Children’s Centres in earlier work in the programme of research), 80% power (2-sided 5% significance), an intraclass correlation coefficient of 0.05 (obtained from interviews with parents in Children’s Centres in earlier work in the programme of research) and that outcomes are assessed on 20 families per Children’s Centre. This gives a total of 33 Children’s Centres. In order that all study coordinating centres recruit the same number of Children’s Centres we will recruit a total of 36 Children’s Centres (9 per study coordinating centre), which will also allow for a potential drop-out of one centre per study arm. In order to achieve outcome data on 20 families per Children’s Centre we will aim to recruit 30 families per Children’s Centre; a total of 1080 families.

# Qualitative data

Data from the interviews with Children’s Centre managers and staff will be managed using a qualitative data management package e.g. QSR NVIVO. The transcripts will be read and re-read to identify themes emerging on the barriers and facilitators and implications for practice and these will be documented. Framework Analysis^19^ will be used. The initial framework will be developed and agreed by researchers from the 4 centres and lay research advisors using data from 8 interviews representing a range of Children’s Centres. This framework will be used by a researcher based in Bristol to undertake the analysis of the rest of the interviews. The framework and analysis will be independently checked in Nottingham and the final framework agreed for analysis of the remaining data. The findings will be reported using anonymised quotations. Data from the telephone contacts and face-face contacts between Children’s Centres and researchers will be analysed manually using content analysis after categorisation into main sub-headings^20, 21^. A thematic analysis will then be conducted.

### Assessment of efficacy

Unless stated otherwise, all comparisons will be made between I_1_ vs C; I_2_ vs C and I_1_ vs I_2_ arms.

### Primary endpoint

Family participants

The proportion of families who have a fire escape plan will be compared between treatment arms by estimating odds ratios and 95% CI.

### Secondary endpoints

1. The proportion of families with smoke alarms fitted and working on every level will be compared between treatment arms by estimating odds ratios and 95% CI;
2. The proportion of families who report fire-setting or match play by their children will be compared between treatment arms by estimating odds ratios and 95% CI;
3. The proportion of families who have a bedtime routine for preventing fires will be compared between treatment arms by estimating odds ratios and 95% CI;
4. The proportion of families who take part in smoking cessation courses/support will be compared between treatment arms by estimating odds ratios and 95% CI;
5. Scores of knowledge of the causes of fires in the home will be compared between treatment arms using random effects linear regression to estimate a mean difference in score plus 95%CI;
6. The proportion of families satisfied with the home safety information provided by Children’s Centre will be compared between treatment arms by estimating odds ratios and 95% CI;
7. Comparison of families’ resource-use and expenditure in the I_1_ and I_2_ arms incurred as a result of the intervention will be compared using incremental cost effectiveness ratios.

Children’s Centres as participants

1. The proportion of Children’s Centres providing information and advice on the prevention of fire-related injury including fire escape planning, smoke alarms, preventing fire setting or match play, use of bedtime routines for preventing fires, smoking cessation and referral to smoking cessation services. These will be compared between treatment arms by estimating OR and 95% CI.
2. The identification of barriers and facilitators to Children’s Centres implementing the IPB (only in Children’s Centres in the I_1_ and I_2_ arms) (qualitative data analysis);
3. Resource-use and expenditure incurred as a result of the intervention in the I_1_ and I_2_ arms will be compared using incremental cost effectiveness ratios;
4. The success of the process of implementing the IPB in Children’s Centres (using telephone and face-face contact during the intervention period (qualitative data analysis) and the implementation measurement tool that will be developed based on the PARIHS framework)^16^ (quantitative data which will be compared between treatment arms using OR and 95% CI for binary or ordinal data and linear regression for continuous data, if assumptions are met).
5. The demographic characteristics of participating families compared with those in the catchment area of Children’s Centres using χ^2^ tests and t-tests, or non-parametric tests as appropriate.

### Assessment of safety

No adverse events are anticipated. Should an adverse event occur the Chief Investigator will be informed who will also report this to the TSC.

### Procedures for missing, unused and spurious data

A complete case analysis will be undertaken. If any Children’s Centres are lost to follow-up we will consider a range of approaches to undertaking sensitivity analyses to assess the robustness of the findings.

### Definition of populations analysed

Full Analysis: the full analysis set will be all families on whom a follow up assessment of the primary outcome measure is available. Every effort will be made to keep in contact with families, even if they move house to an area covered by a different Children’s Centre, to minimise losses to follow-up.

# ADVERSE EVENTS

The occurrence of adverse events as a result of participation within this study is not anticipated. We believe the risks for participants in this study are minimal, indeed this trial aims to increase participants’ safety. However, should an adverse event occur this will be recorded and reported to the Chief Investigator who will also report these to the TSC. Any participant who experiences an adverse event who wishes to be withdrawn from the study will be allowed to do so. Any adverse events will also be reported in the final study report.

# ETHICAL AND REGULATORY ASPECTS

## ETHICS COMMITTEE AND REGULATORY APPROVALS

The trial will not be initiated before the protocol, informed consent forms and participant information sheets have received approval / favourable opinion from the NHS Research Ethics Committee (NRES), the University of the West of England, Bristol Research Ethics Committee, and the respective National Health Service (NHS) Research & Development (R&D) department, if appropriate. Should a protocol amendment be made that requires REC approval, the changes in the protocol will not be instituted until the amendment and revised informed consent forms and participant information sheets (if appropriate) have been reviewed and received approval / favourable opinion from the REC and R&D departments. A protocol amendment intended to eliminate an apparent immediate hazard to participants may be implemented immediately providing that the REC are notified as soon as possible and an approval is requested. Minor protocol amendments only for logistical or administrative changes may be implemented immediately; the REC will be informed.

The trial will be conducted in accordance with the ethical principles that have their origin in the Declaration of Helsinki, 2008; the principles of Good Clinical Practice, and the Department of Health Research Governance Framework for Health and Social care, 2005.

##

## INFORMED CONSENT AND PARTICIPANT INFORMATION

The process for obtaining participant informed consent will be in accordance with the REC guidance, Good Clinical Practice (GCP) and any other regulatory requirements that might be introduced. The researcher and the participant or other legally authorised representative shall both sign and date the Informed Consent Form before the person can participate in the trial. This may be in person or via post when a copy of the signed and dated ICF will be sent to the parent(s) (family participants) or Children’s Centre (Children’s Centre participants).

The participant will receive a copy of the signed and dated forms and the original will be retained in the trial master file at each study coordinating centre.

The decision regarding participation in the study is entirely voluntary. The researcher will emphasise to all participants (families and Children’s Centres) that consent regarding study participation may be withdrawn at any time without penalty or affecting the quality or quantity of the services provided by the Children’s Centre (for family participants), or loss of benefits to which the participant is otherwise entitled. No trial-specific interventions will start before informed consent has been obtained.

The investigator will inform the participant of any relevant information that becomes available during the course of the study, and will discuss with them, whether they wish to continue with the study. If applicable they will be asked to sign revised consent forms.

If the Informed Consent Form is amended during the study, the investigator shall follow all applicable regulatory requirements pertaining to approval of the amended Informed Consent Form by the REC and use of the amended form (including for ongoing participants).

## RECORDS

### Source documents: Case Report Forms

Each Children’s Centre and family will be assigned a unique identity code (UIC) to facilitate the conduct and analysis of the trial. These will be used on CRFs, other trial documents and electronic databases. The Children’s Centre code will consist of: two letters at the start to identify the study coordinating centre (PI’s initials), followed by the first three letters of the Children’s Centre name and two digits will be the Children’s Centre’s trial number. The families’ UIC will consist of two letters at the start to identify the study coordinating centre (PI’s initials), then two digits for the Children’s Centre’s trial number, four digits for families’ trial number, followed by two letters for their initials (of first and last names).

Recruitment Logs

Recruitment logs will serve as a separate confidential record of the participants’ details. This will permit identification of all participants enrolled in the trial, in accordance with regulatory requirements and for follow-up, as required. Two logs will be created; one for Children’s centres and one for family participants.

An Access database recruitment log of all Children’s Centres invited to take part will be created with the following information in it:

- Unique Identity Code (UIC)
- Children’s Centre Name
- Study coordinating centre
- PCT
- Children’s Centre address
- Children’s Centre telephone number/other contact details
- Lead organisation
- Date approached to take part
- Date consent form received
- Would like book voucher at end of study
- Date baseline questionnaire received
- Recruited to trial
- Date randomised
- Treatment arm code
- Date intervention started (if relevant)
- Which staff or health professionals are delivering the intervention
- Names of parents attending each intervention session
- Date intervention completed (if relevant)
- Contact names: eg., Children’s Centre managers/contact person for implementing IPB; health visitors; local Fire Safety Officer
- Other relevant information.

An Access database recruitment log of all families invited to take part will be created with the following information in it:

- Unique Identity Code (UIC)
- Date approached to take part
- Date agreed to participate
- Date consent form received
- Date recruited to trial
- Date baseline questionnaire received
- Would like £5 voucher for baseline questionnaire
- Treatment arm code
- Date baseline questionnaire completed
- Date first intervention received, as recorded by Children’s Centre
- Date follow-up questionnaire received
- Date follow-up questionnaire completed
- Would like £5 voucher for follow-up questionnaire
- Other relevant information.

In addition the following data will be collected and stored on a separate Access

database: families’ UIC and parent(s) name, address, telephone and other relevant contact details, eg., date follow-up questionnaire due, preferred format for administration of follow up questionnaire, email address.

Questionnaires

Completed questionnaires will form the CRFs. These will be treated as confidential documents and held securely. No identifiable information will be recorded on the questionnaire and they will not be stored with any identifiable data (e.g. consent forms). Access to CRFs shall be restricted to those personnel approved by the Chief or local Principal Investigator and recorded on the ‘Trial Delegation Log’.

Interview data

Interviews will be conducted by researchers from each of the four study coordinating centres. With the permission of the interviewees, interviews will be digitally audio-recorded. If recordings require transmission between study coordinating centres they will be encrypted and emailed or loaded onto a password protected secure portal (the NIHR portal) for access by other study coordinating centres. They will be anonymised and stored with the research data as source data. The transcribed interviews will be treated as confidential documents and held securely with no identifiable information on the transcripts. These will be stored on a secure password protected University or NHS server. At the end of the study, data from all centres will be archived by the Nottingham centre for a period of seven years as set out in the University of Nottingham’s Code of Research Conduct.

### Direct access to source data / documents

All source documents shall be made available at all times for review by the Chief Investigator, Sponsor’s designee and inspection by relevant ethical regulatory authorities.

Source documents shall be filed at the investigator’s site and may include but are not limited to consent forms, questionnaires, interview transcripts and field notes. A CRF may also completely serve as its own source data. Only study staff shall have access to study documentation other than the regulatory requirements listed below.

## DATA PROTECTION

All trial staff and investigators will endeavour to protect the rights of the trial’s participants to privacy and informed consent, and will adhere to the Data Protection Act, 1998. The CRFs will only collect the minimum required information for the purposes of the trial. CRFs will be held securely, in a locked room, or locked cupboard or cabinet. Access to the information will be limited to the trial researchers and investigators and relevant regulatory authorities (see above). Computer held data, including trial databases, will be held by each study coordinating centre on secure, password protected University or NHS server and will be backed up as a minimum every 24 hours. Personal laptops will not be used. Where University of NHS laptops are used, these will be password protected and data will be transferred to the University/NHS server as soon as possible.

# QUALITY ASSURANCE & AUDIT

## INSURANCE AND INDEMNITY

Insurance and indemnity for trial participants and trial staff (employed by the NHS or holding NHS honorary research or clinical contracts) is covered within the NHS Indemnity Arrangements for clinical negligence claims in the NHS, issued under cover of HSG (96) 48. There are no special compensation arrangements, but trial participants may have recourse through the NHS complaints procedures.

The University of Nottingham has taken out an insurance policy to provide indemnity in the event of a successful litigious claim for proven non-negligent harm.

## TRIAL CONDUCT

Trial conduct will be subject to systems audit of the Trial Master File and the Investigator Site File at each of the four study coordinating centres. These will include essential documents; permissions to conduct the trial; Site Delegation Log; CVs of trial staff and training received; local document control procedures; consent procedures and recruitment logs; adherence to procedures defined in the protocol (e.g. inclusion / exclusion criteria, correct randomisation, timeliness of visits); adverse event recording and reporting and accountability of trial materials.

The Trial Managers, or where required, a nominated designee of the Sponsor, shall carry out a site systems audit at least yearly and an audit report shall be made to the Trial Steering Committee.

## TRIAL DATA

Monitoring of trial data shall include confirmation of informed consent; source data verification; data storage and data transfer procedures; local quality control checks and procedures, back-up and disaster recovery of any local databases and validation of data manipulation.

Entries on baseline and follow up questionnaires will be verified by inspection. Data entry, checking, correcting and manipulation will be undertaken in accordance with the data management standard operating procedure.

Trial data and evidence of monitoring and systems audits will be made available for inspection by REC as required.

## RECORD RETENTION AND ARCHIVING

In compliance with the ICH/GCP guidelines, regulations and in accordance with the University of Nottingham Research Code of Conduct, the Chief or local Principal Investigator will receive and maintain all records and documents regarding the conduct of the study after the completion of the trial. These will be retained for at least 7 years or for longer if required. If the responsible investigator is no longer able to maintain the study records, a second person will be nominated to take over this responsibility.

The Trial Master File and trial documents held by the Chief Investigator on behalf of the Sponsor shall be finally archived at secure archive facilities at the University of Nottingham. This archive shall include all trial databases and associated meta-data encryption codes.

## DISCONTINUATION OF THE TRIAL BY THE SPONSOR

The Sponsor reserves the right to discontinue this trial at any time for failure to meet expected enrolment goals, for safety or any other administrative reasons. The Sponsor shall take advice from the Trial Steering Committee in making this decision.

## STATEMENT OF CONFIDENTIALITY

Individual participant information obtained as a result of this study is considered confidential and disclosure to third parties is prohibited with the exceptions noted above. Participant confidentiality will be further ensured by using identification code numbers to correspond to trial data in the computer files.

Data generated as a result of this trial will be available for inspection on request by the University of Nottingham representatives, the REC, local R&D Departments and the regulatory authorities.

# PUBLICATION AND DISSEMINATION POLICY

Investigators, advisors and researchers will contribute towards drafting the papers reporting the findings of the main studies and all will be named authors on those papers, providing they fulfil the Vancouver criteria for authorship.

Investigators, advisors and researchers wishing to analyse and report other findings from the studies can do so on the agreement of the other investigators, and the study team will agree authorship for these papers, subject to the Vancouver criteria for authorship.

The investigators regard active, widespread dissemination of outputs from this research as essential. The findings of this research should reflect the multi-disciplinary nature of unintentional injury and its prevention, with the information generated by the research being made available to the range of relevant stakeholders in accessible and usable forms, ensuring that it informs policy and practice as well as adding to knowledge generally. Outputs will be targeted towards policy-makers, academic researchers, practitioners, prevention specialists and study participants.

The investigators will seek to publish the findings of the work, including methodological issues, in peer-reviewed journals, at conferences (including the biannual world injury prevention conferences), in specialist publications aimed at specific practitioner audiences and through a dedicated website. The website will give easy worldwide access to descriptions of work in progress, links to project outputs, and information about the researchers. It will be written in jargon-free language suitable for a general audience.

# USER AND PUBLIC INVOLVEMENT

At least one lay research advisor is involved as an active member of the steering group and has been since its inception and will continue to be up to the final report writing and writing papers for publication.

Lay advisors are working with researchers to develop the trial interventions, the study tools and processes, to recruit and work with parents or carers with young children to pilot and modify tools and processes.

# STUDY FINANCES

### Funding source

This study is funded by the National Institute for Health Research.

### Participant stipends and payments

Family participants in the trial will receive a £5 gift voucher for local stores on receipt of each of the baseline and follow-up completed questionnaires. Participating Children’s Centre will be offered a £25 book voucher. No other payments will be made to participants in this study.

# SIGNATURE PAGES

Signatory to Protocol:

**Chief Investigator:** Denise Kendrick

Signature:__________________________________

Date: ___________

# REFERENCES

- - 1. Office for National Statistics. Mortality Statistics: Childhood, infant and perinatal. Series DH3 No 36. London: Office for National Statistics, 2005.
    2. Mulvaney C, Kendrick D, Towner E, Brussoni M, Hayes M, Powell J, Robertson S, Ward H. Fatal and non-fatal injuries in England 1995-2004: time trends and inequalities by age, sex and area deprivation. *Journal of Public Health,* 2008; 31(1): 154-161.
    3. Edwards P, Roberts I, Green J et al. Deaths from injury in children and employment status in family: analysis of trends in class specific death rates. BMJ 2006;333:119–21.
    4. UNICEF. A league table of child deaths by injury in rich nations. Florence: Innocenti Research Centre, 2001. Report No. 2.
    5. Joffe AR, Lalani A. Injury Admissions to Pediatric Intensive Care Are Predictable and Preventable: A Call to Action. *Journal of Intensive Care Medicine* 2006;21(4):227-234.
    6. Department of Health. *Saving Lives: Our Healthier Nation*. London: The Stationery Office, 1999.
    7. Department of Health. *Choosing health: making healthier choices easier* (CM 6374). London: Department of Health, 2004.
    8. Department of Health. *Preventing Injury - priorities for action*. A report from the Accidental Injury Task Force to the Chief Medical Officer. London: Department of Health, 2002.
    9. Department of Health, Department for Education and Skills. *National Service Framework for children, young people and maternity services: Core standards*. London: Department of Health, Department for Education and Skills, 2004.
    10. Department for Children Schools and Families. Staying Safe: Action Plan. Nottingham: DCSF, 2008:81.
    11. Department for Education and Skills. *Every child matters: Change for children*. Nottingham: Department for Education and skills, 2004.
    12. Department for Education and Skills. *Working together to safeguard children*. London: The Stationery Office, 2006.
    13. Department of Health. *Healthy Lives, Healthy People*. London: Department of Health, 2010.
    14. The Audit Commission, Health Care Commission. *Better safe than sorry: preventing unintentional injury to children*. London: The Audit Commission, 2007.
    15. Edwards PJ, Roberts I, Clarke MJ, DiGuiseppi C, Wentz R, Kwan I, Cooper R, Felix LM, Pratap S. Methods to increase response to postal and electronic questionnaires. Cochrane Database of Systematic Reviews 2009, Issue 3. Art. No.: MR000008. DOI: 10.1002/14651858.MR000008.pub4. Editorial Group: [Methodology Review Group](http://www2.cochrane.org/reviews/en/subtopics/33.html); This version first published online: October 20. 2003; Last assessed as up-to-date: December 10. 2008.
    16. Rycroft-Malone J: The PARIHS framework – a framework for guiding the implementation of evidence-based practice. *Journal of Nursing Care Quality* 2004, 19:297-304.
    17. Helfrich, CD, Li Yu-Fang, Sharp ND, Sales AE. Organizational readiness to change assessment (ORCA): Development of an instrument based on the Promoting Action on Research in Health Services (PARIHS) framework. *Implementation Science* 2009, 4:38 doi:10.1186/1748-5908-4-38.
    18. Kendrick D, Coupland C, Mulvaney C, Simpson J, Smith S, Sutton A, et al. Home safety education and provision of safety equipment for injury prevention. *Cochrane Database Systematic Review* 2007; Issue 1 (Art. No.:CD005014): DOI: 10.1002/14651858.CD005014.pub2.
    19. Ritchie J, Spencer L. Qualitative data analysis for applied policy research. In: Bryman A, Burgess R, editors. *Analyzing Qualitative Data*. Oxford: Routledge, 1994.
    20. Morgan D. Qualitative content analysis: a guide to paths not taken. *Qualitative Health Research* 1993; 3: 112–121.
    21. Pope C, Ziebland S. & Mays N. Analysing qualitative data. In *Qualitative Research in Health Care* (Pope C. & Mays N., eds), BMJ Books, London, pp. 75–88, 2000.

# STUDY FLOWCHART
